# Supplementary material for: Spatial tumor immune heterogeneity facilitates subtype co-existence and therapy response in pancreatic cancer
Source: Nat Commun. 2025 Jan 6;16:335. doi: 10.1038/s41467-024-55330-7 (PMC11704331; doi:10.1038/s41467-024-55330-7)
Supplement: Supplementary file 1 — Supplementary Information [file 41467_2024_55330_MOESM1_ESM.pdf]

# Spatial tumor immune heterogeneity facilitates subtype co-existence and therapy response in pancreatic cancer

## SUPPLEMENTARY INFORMATION – TABLE OF CONTENTS

### Supplementary Figures

Supplementary Figure 1. Association of cJUN with GATA6 in PDAC.
Supplementary Figure 2. JUNB restricts invasiveness and pro-inflammatory signaling.
Supplementary Figure 3. JUNB antagonizes BL inflammatory signaling and cJUN.
Supplementary Figure 4. cJUN overexpression enhances TNF-α$^{+}$ macrophage recruitment.
Supplementary Figure 5. TNF-α and JUNB repression shapes TiME heterogeneity.
Supplementary Figure 6. TNF-α expression is associated with T cell exclusion.

### Supplementary Tables

Supplementary Table 1. Antibody details
Supplementary Table 2. Primer sequences and siRNAs
Supplementary Table 3. Directly JUNB-repressed Genes in CAPAN1
Supplementary Table 4. JUNB repression signature

## SUPPLEMENTARY FIGURES

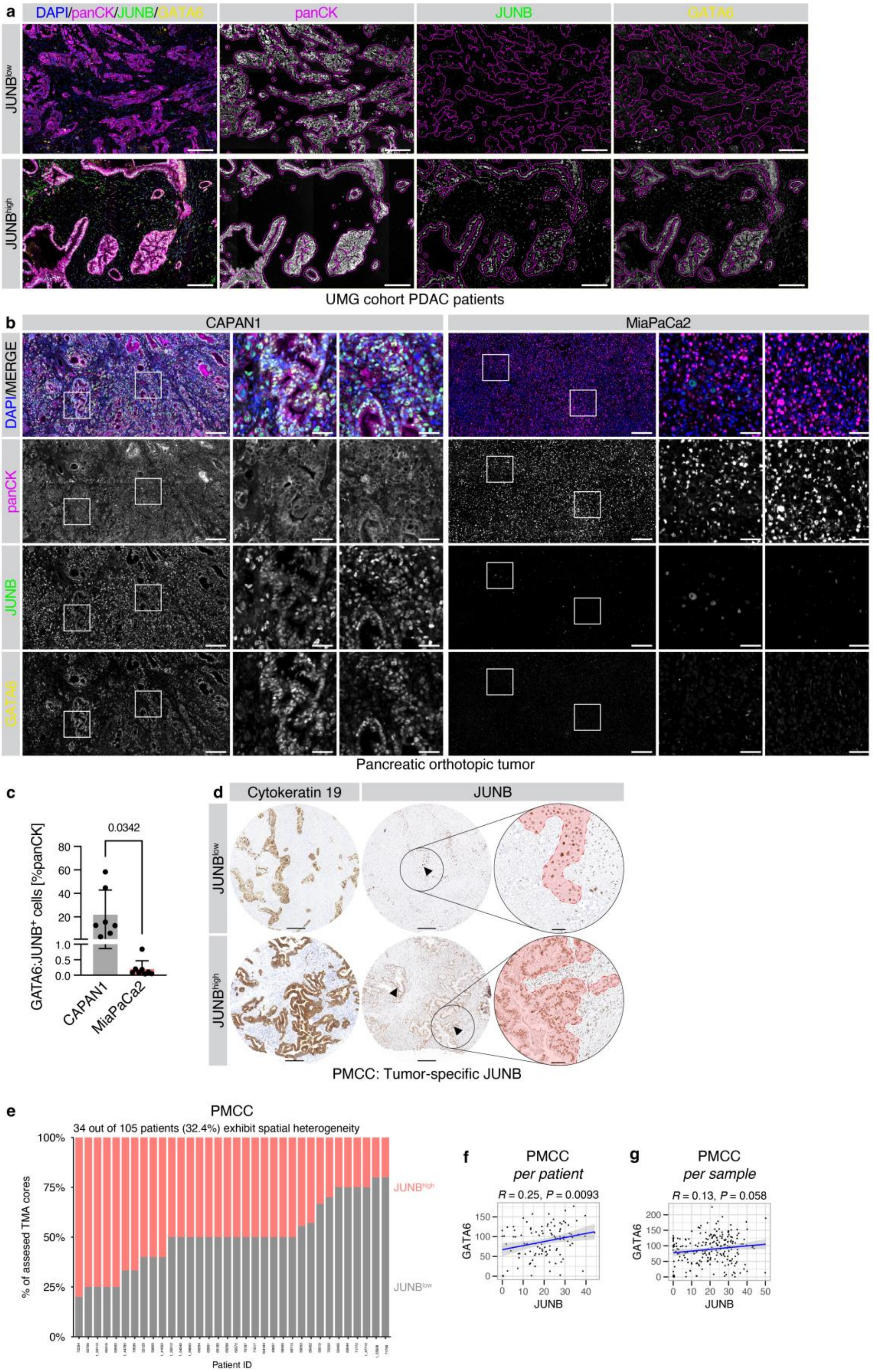


**Supplementary Figure 1.** *Legend on following page*.

**Supplementary Figure 1. Association of cJUN with GATA6 in PDAC. a**, IF for JUNB, GATA6, and pan-cytokeratin (panCK) in resection tissue of therapy-naive PDAC patients of the University Medical Center Göttingen (UMG) cohort at representative region with high, intermediate, and low epithelial JUNB expression, as in **Fig. 1e**. Epithelial area is overlayed on greyscale images in magenta, based on $panCK^+$ cell classification by QuPath. In the overlay, blue: DAPI, green: JUNB, magenta: panCK, yellow: GATA6. Scale bar: 200 μm. **b**, As in **a**, for orthotopically transplanted CAPAN1 and MiaPaCa2 cells into NMRI-*Foxn1*$^{nu/nu}$ mice. Scale bar: overview, 200 μm; insert, 50 μm. **c**, Quantification of **b** for per-animal average percentage of GATA6:$JUNB^+$ epithelial ($panCK^+$) cells with mean ± s.d. shown. CAPAN1, n=7 animals; MiaPaCa2, n=8 animals. Student's t-test with Welch's correction. **d**-**g**, IHC analysis in 105 PDAC patients of the Princess Margaret Cancer Centre (PMCC) cohort for epithelial JUNB expression. **d**, IHC for cytokeratin 19 (CK19) and JUNB in TMA cores classified as $JUNB^{low}$ and $JUNB^{high}$, indicating tumor-specific $JUNB^+$ cells. Inserts show higher magnification of JUNB staining and CK19-based estimation of epithelial neoplastic area. Scale bar: overview 200 μm; insert 50 μm. **e**, Spatial heterogeneity of tumor-specific JUNB expression within different TMA cores of each patient. **f**,**g**, Correlation of JUNB and GATA6 IHC quantification per patient (**f**) or per TMA core across all patients (**g**). Linear regression with 95% CI, as well as Spearman's *R* and associated *P* value. Source data are provided as a Source Data file.

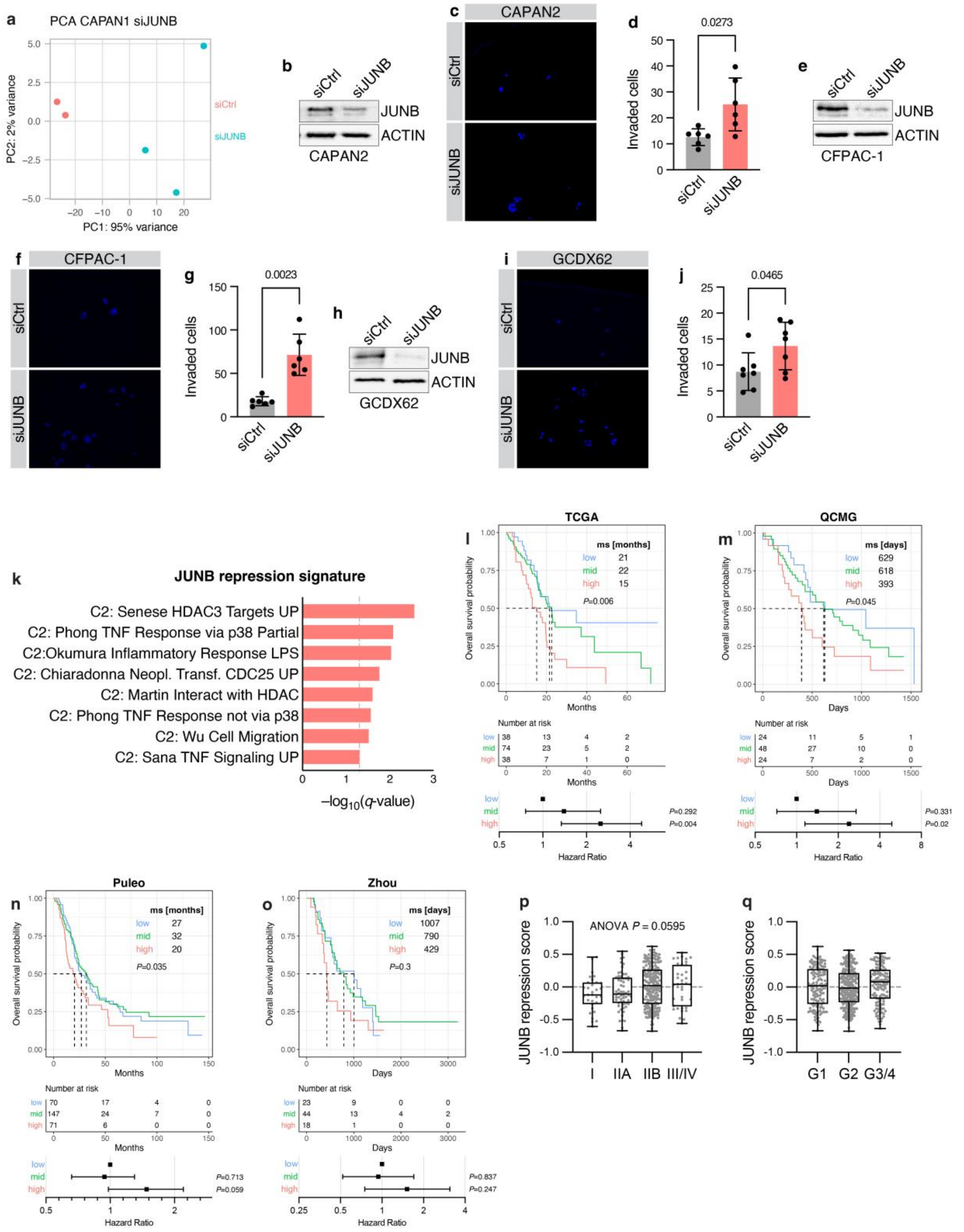

**Supplementary Figure 2. JUNB restricts invasiveness and pro-inflammatory signaling. a**, PCA plot for RNA-seq data performed for JUNB silencing (siJUNB; n=3 biological replicates) or control siRNA (siCtrl; n=2 biological replicates) in CAPAN1. **b-j**, Transwell invasion assay for CAPAN2 (**b-d**), CFPAC-1 (**e-g**), and GCDX62 (**h-j**) with siJUNB or siCtrl.

*Continued on following page.*

**Supplementary Figure 2** *continued.* **b**,**e**,**h**, Immunoblot for JUNB and β-actin after siJUNB or siCtrl in CAPAN2 (**b**), CFPAC-1 (**e**), and GCDX62 (**h**), validating silencing for the invasion assay. n=3 biological replicates. **c**,**f**,**i**, DAPI staining of invaded CAPAN2 (**c**), CFPAC-1 (**f**), or GCDX62 (**i**) cells. Scale bar 100 µm. **d**,**g**,**j**, Quantification of **c**,**f**,**i**, for number of invaded cells. Average counts per FOV with mean ± s.d. shown. **d**,**g**, n=6 inserts from n=3 independent experiments. **j**, n=7 inserts from n=4 independent experiments. Student's t-test with Welch's correction. **k**, Gene ontology analysis of the JUNB repression signature (see **Fig. 2j**) with $-\log_{10}$(*q*-value) indicated. Hallmark (H) and curated (C2) signature collections of the Molecular Signature Database (MSigDB) are shown. **l-o**, Overall survival, numbers at risk, and hazard ratio in TCGA (**l**, n=150), QCMG (**m**, n=96), Puleo (**n**, n=288) and Zhou (**o**, n=85) patients stratified by JUNB repression signature score. Top: Kaplan-Meier survival analysis for the lower/upper quartiles and mid group for JUNB repression signature scores. Median survival (ms) is indicated. Log-rank test. Bottom: Cox proportional hazard. Hazard ratio (to lower quartile) with 95% CI. *P* values are shown right. **p**,**q**, JUNB repression signature scores in AJCC stages in TCGA, QCMG, and Zhou cohorts (**p**) and pathological grading for TCGA, QCMG, and Puleo cohorts (**q**) combined. Source data are provided as a Source Data file.

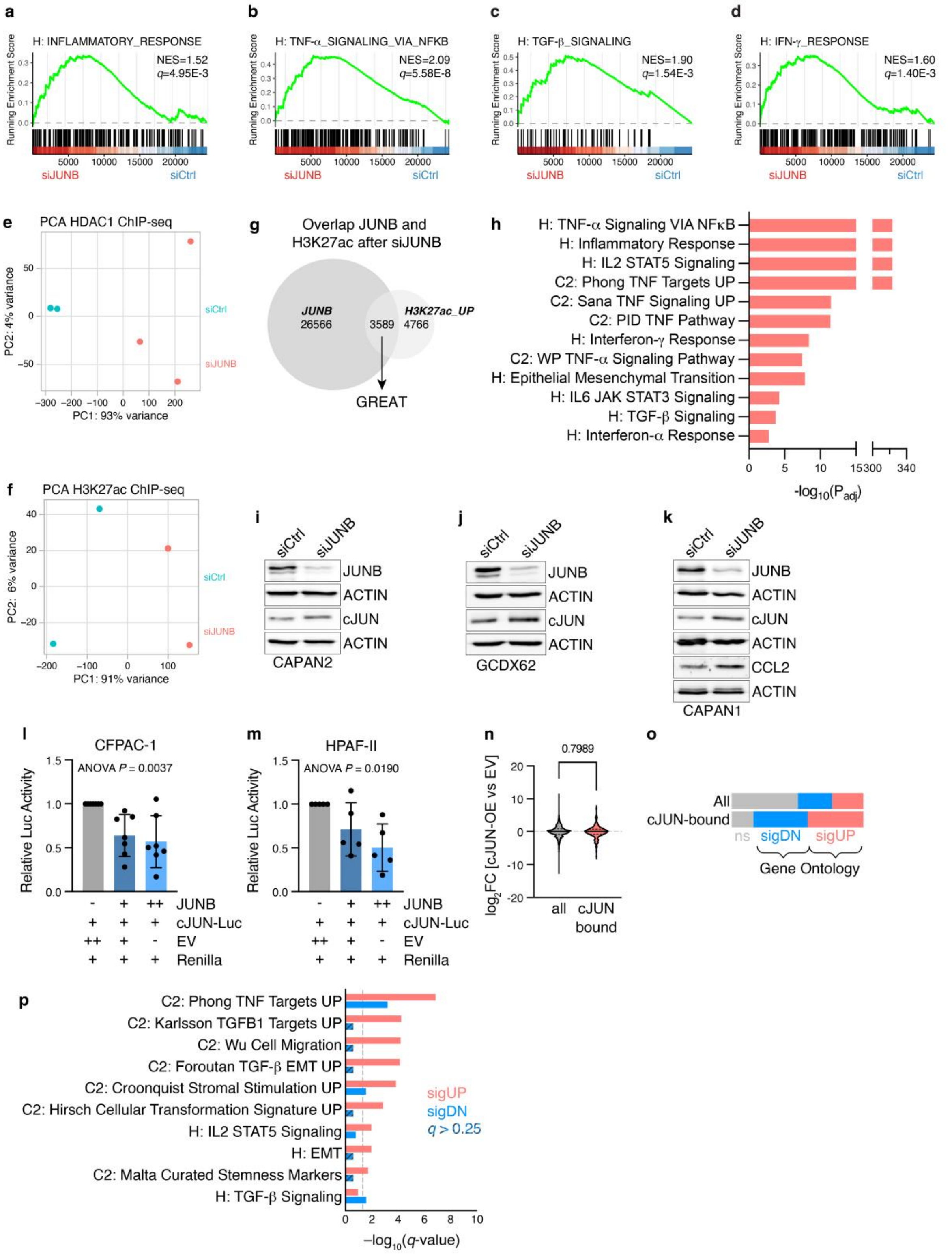


**Supplementary Figure 3. JUNB antagonizes BL inflammatory signaling and cJUN. a-d**, Gene set enrichment analysis plots for "inflammatory response" (**a**), "TNF-α signaling via NFκB" (**b**), "TGF-β signaling" (**c**) and "IFN-γ response" (**d**) Hallmark signatures of the MSigDB for siJUNB versus siCtrl in CAPAN1 cells. Normalized enrichment score (NES) and FDR $q$ value are indicated.

*Continued on following page.*

**Supplementary Figure 3** *continued.* **e**,**f**, PCA plot for ChIP-seq data performed for HDAC1 (**e**) and H3K27ac (**f**) after JUNB silencing (siJUNB) or control siRNA (siCtrl) in CAPAN1. **e**, siCtrl, n=2 biological replicates; siJUNB, n=3 biological replicates. **f**, n=2 biological replicates. **g**, Overlap of JUNB binding regions in control cells and regions where H3K27ac is significantly gained upon siJUNB ("H3K27ac_UP"). **h**, GREAT analysis of the overlapping region of **g** with $-\log_{10}(P_{adj})$ for binomial test indicated. Hallmark (H) and curated (C2) signature collections of the Molecular Signature Database (MSigDB) are shown. **i**,**j**, Representative immunoblot for JUNB, cJUN, and β-actin in CAPAN2 (**i**) and GCDX62 (**j**) after siJUNB or siCtrl. **i**, n=2 biological replicates; **j**, n=3 biological replicates. **k**, Representative immunoblot for JUNB, cJUN, CCL2 and β-actin in CAPAN1 after siJUNB or siCtrl. n=2 biological replicates. **l**,**m**, Dual-luciferase reporter assay for cJUN promoter firefly luciferase constructs in CFPAC-1 (**l**) and HPAF-II (**m**) cells transfected with varying concentrations of JUNB overexpression plasmids (or EV controls), together with *Renilla* luciferase control and firefly luciferase (Luc) reporters. Relative Luc activity to control with mean ± s.d. shown. One-way ANOVA. **l**, n=7 biological replicates. **m**, n=5 biological replicates. **n**-**p**, Integration of RNA-seq data performed in three biological replicates for overexpression of cJUN (cJUN-OE) or empty vector (EV) control in GCDX62 with ChIP-seq for cJUN. **n**, Violin plot of $\log_2$ fold change (FC) in cJUN-OE RNA-seq data for all (n=24.118) or cJUN-bound (n=224) genes. Median and quartiles are indicated. **o**, As in **n**, showing the number of genes that display a significant upregulation (sigUP) or downregulation (sigDN), or no significant change (ns). **p**, Gene ontology analysis of significantly upregulated (red) or downregulated (blue), cJUN-bound genes following cJUN-OE with $-\log_{10}(q\text{-value})$ indicated. Hallmark (H) and curated (C2) signature collections of the MSigDB are shown. Not enriched pathways ($q$-value>0.25) are indicated by striped bars. Source data are provided as a Source Data file.

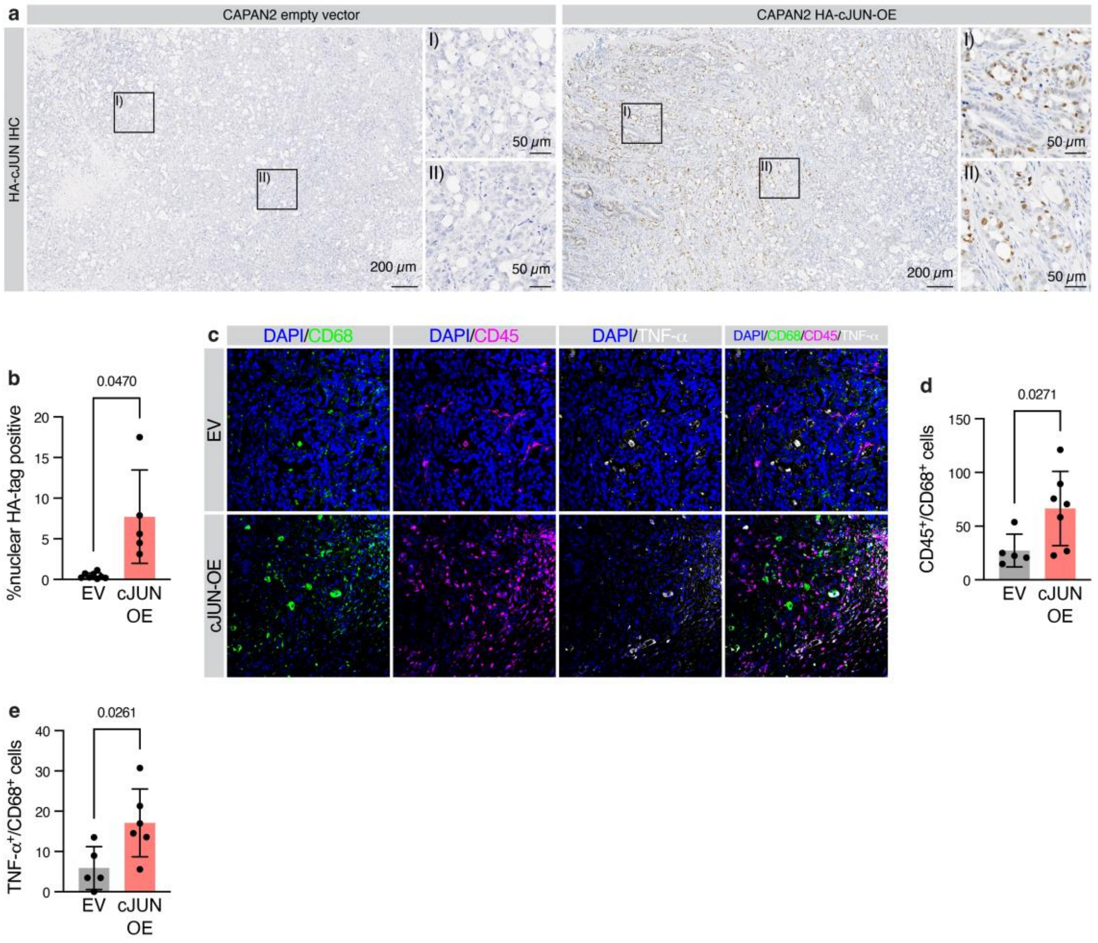


**Supplementary Figure 4. cJUN overexpression enhances TNF-α$^{+}$ macrophage recruitment.** **a**-**e**, NMRI-*Foxn1*$^{nu/nu}$ mice were orthotopically transplanted with CAPAN2 cells with stable HA-tagged cJUN-OE or EV control. **a**, IHC for the HA tag of cJUN in orthotopically transplanted CAPAN2 HA-cJUN-OE and EV tumors. Higher magnification insert areas are indicated. Scale bar: overview area, 200 μm; insert, 50 μm. **b**, Quantification of **a** for nuclear HA-tag-positive cells relative to the total number of detected cells with mean ± s.d. shown. EV, n=8 animals; cJUN-OE, n=5 animals. **c**, Representative IF staining for CD68, CD45, and TNF-α in orthotopically transplanted CAPAN2 HA-cJUN-OE and EV tumors. Scale bar: 50 μm. **d**,**e**, Quantification of **c** for CD45/CD68 (**d**) and TNF-α/CD68 (**e**) double-positive cells. Per-animal average counts per FOV with mean ± s.d. shown. **d**, EV, n=5 animals; cJUN-OE, n=7 animals. **e**, EV, n=5 animals; cJUN-OE, n=6 animals. **b**,**d**,**e**, Student’s t-test with Welch’s correction. Source data are provided as a Source Data file.

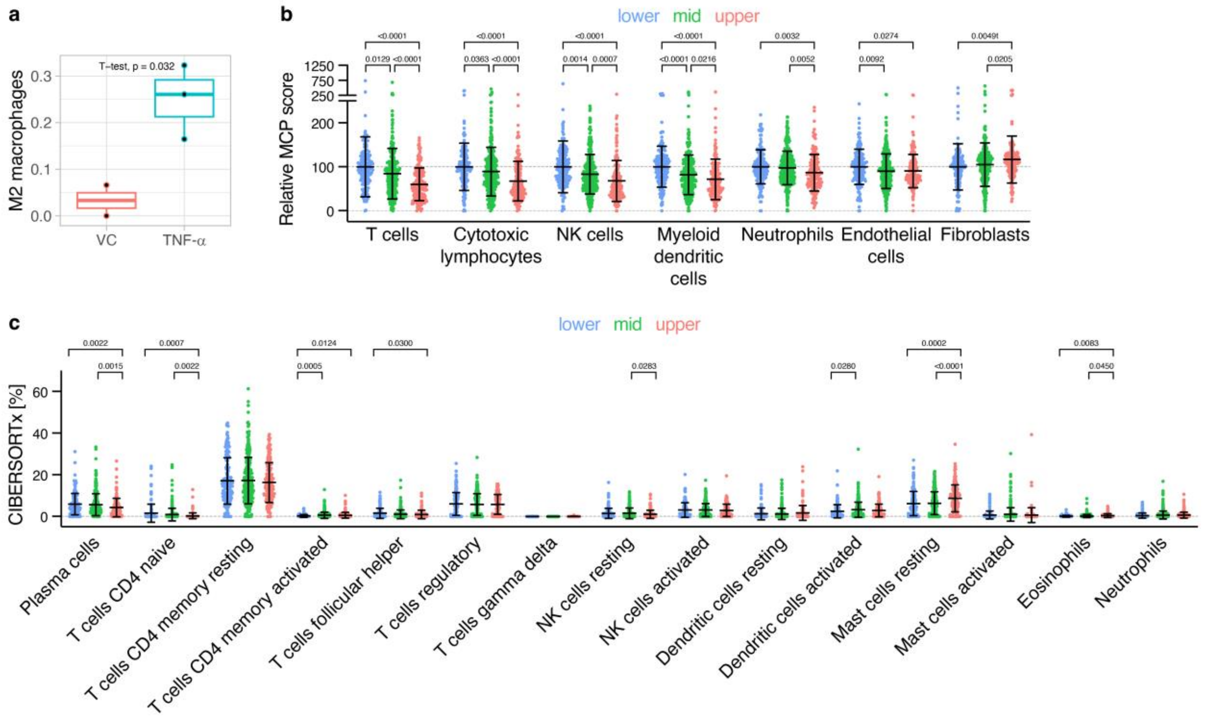


**Supplementary Figure 5. TNF-α and JUNB repression shapes TiME heterogeneity. a**, CIBERSORTx analysis for M2 macrophages in stromal compartment of virtually microdissected RNA-seq data of orthotopically transplanted CAPAN1 tumors treated with TNF-α or VC for three weeks (see **Fig. 5**). VC, n=2 tumors; TNF-α, n=3 tumors. Student's t-test with Welch's correction. Boxplots show 25$^{th}$ to 75$^{th}$ percentile with median as box and highest and lowest value in 1.5 times interquartile range as whiskers. **b**, Relative MCPcounter scores for the indicated lineages in n=652 patients of the TCGA, QCMG, Puleo, and Zhou cohorts, separated into quartiles based on the JUNB repression signature score (as in **Figure 2k**,**l**). MCPcounter scores were min-max normalized and standardized to the mean of the lower JUNB repression signature score group for merging of the different cohorts. Mean ± s.d. shown. **c**, As in **b**, but applying CIBERSORTx for deconvolution. Mean ± s.d. for CIBERSORTx percentages shown. Source data are provided as a Source Data file.

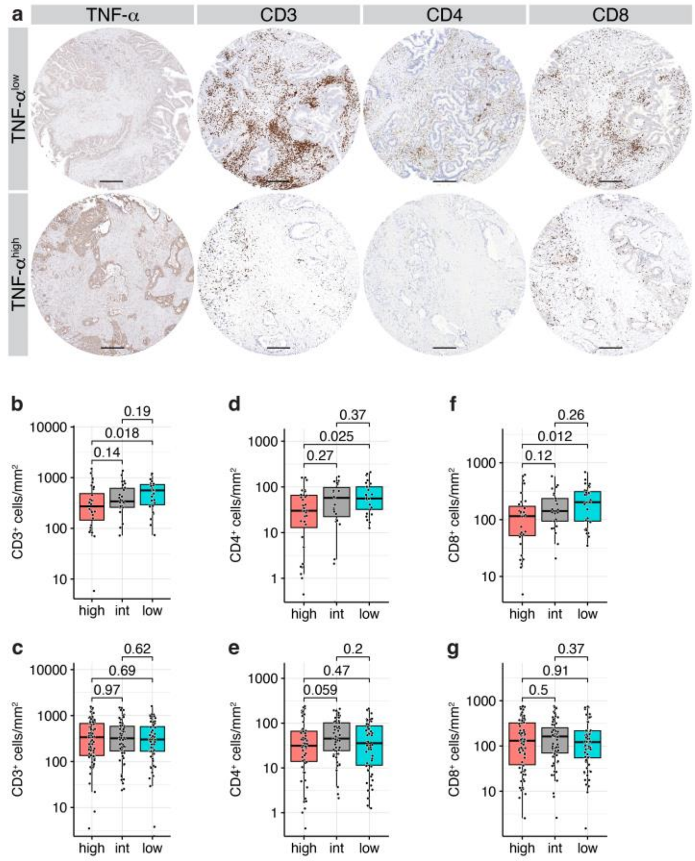


**Supplementary Figure 6. TNF-α expression is associated with T cell exclusion.** **a**-**g**, IHC analysis in 105 PDAC patients for TNF-α expression. **a**, IHC for TNF-α, CD3, CD4, and CD8 in cores classified as TNF-$\alpha^{low}$ and TNF-$\alpha^{high}$. Sale bar 200 μm. **b**-**g**, Quantification of **a**, for CD3 (**b**,**c**), CD4 (**d**,**e**), and CD8 (**f**,**g**) in TNF-$\alpha^{low}$, TNF-$\alpha^{intermediate}$ (TNF-$\alpha^{int}$), and TNF-$\alpha^{high}$ expression per patient (**b**,**d**,**f**) and per TMA core across all patients (**c**,**e**,**g**). **b**, TNF-$\alpha^{high}$, n=32 patients; TNF-$\alpha^{int}$, n=26 patients; TNF-$\alpha^{low}$, n=30 patients. **c**, TNF-$\alpha^{high}$, n=86 cores; TNF-$\alpha^{int}$, n=82 cores; TNF-$\alpha^{low}$, n=69 cores. **d**, TNF-$\alpha^{high}$, n=32 patients; TNF-$\alpha^{int}$, n=24 patients; TNF-$\alpha^{low}$, n=25 patients. **e**, TNF-$\alpha^{high}$, n=62 cores; TNF-$\alpha^{int}$, n=60 cores; TNF-$\alpha^{low}$, n=64 cores. **f**, TNF-$\alpha^{high}$, n=31 patients; TNF-$\alpha^{int}$, n=25 patients; TNF-$\alpha^{low}$, n=31 patients. **g**, TNF-$\alpha^{high}$, n=78 cores; TNF-$\alpha^{int}$, n=77 cores; TNF-$\alpha^{low}$, n=68 cores. Boxplots show 25$^{th}$ to 75$^{th}$ percentile with median as box and highest and lowest value in 1.5 times interquartile range as whiskers. Source data are provided as a Source Data file.

# SUPPLEMENTARY TABLES

## Supplementary Table 1. Antibody details

| Name | Company | Catalog | Dilution/ Amount | Link to manufacture’s info |
|---|---|---|---|---|
| **CD3** | Abcam | ab16669 | 1:200 (IHC) | https://www.abcam.com/products/primary-antibodies/cd3-antibody-sp7-ab16669.html |
| **CD31** | Miltenyi Biotec | 130-110-675 | 1:50 (FC) | https://www.miltenyibiotec.com/DE-en/products/cd31-antibody-anti-human-reafinity-rea730.html#gref |
| **CD326 (EPCAM)** | Miltenyi Biotec | 130-113-268 | 1:10 (FC) | https://www.miltenyibiotec.com/DE-en/products/cd326-epcam-antibody-anti-human-hea-125.html#fitc:100-tests-in-200-ul |
| **CD45** | Miltenyi Biotec | 130-113-124 | 1:10 (FC) | https://www.miltenyibiotec.com/DE-en/products/cd45-antibody-anti-human-5b1.html#conjugate=viogreen:size=100-tests-in-200-ul |
| **CD45** | BD Pharmingen | 550539 | 1:50 (IF) | https://www.bdbiosciences.com/en-us/products/reagents/flow-cytometry-reagents/research-reagents/single-color-antibodies-ruo/purified-rat-anti-mouse-cd45.550539 |
| **CD68** | Abcam | ab955 | 1:50 (IF) | https://www.abcam.com/cd68-antibody-kp1-ab955.html |
| **CD8** | Abcam | ab217344 | 1:1000 (IHC) | https://www.abcam.com/products/primary-antibodies/cd8-alpha-antibody-epr21769-ab217344.html |
| **cJUN** | Cell Signaling Technology | 9165S | 1:1000 (WB) | https://www.cellsignal.de/products/primary-antibodies/c-jun-60a8-rabbit-mab/9165?site-search-type=Products&N=4294956287&Ntt=9165s&fromPage=plp&_requestid=2228711 |
| **donkey anti-goat IgG Alexa Fluor 568** | Invitrogen | A-11057 | 1:500 (IF) | https://www.thermofisher.com/antibody/product/Donkey-anti-Goat-IgG-H-L-Cross-Adsorbed-Secondary-Antibody-Polyclonal/A-11057 |
| **donkey anti-goat IgG Alexa Fluor 647** | Invitrogen | A32849 | 1:500 (IF) | https://www.thermofisher.com/antibody/product/Donkey-anti-Goat-IgG-H-L-Highly-Cross-Adsorbed-Secondary-Antibody-Polyclonal/A32849 |
| **donkey anti-mouse IgG Alexa Fluor 568** | Invitrogen | A10037 | 1:500 (IF) | https://www.thermofisher.com/antibody/product/Donkey-anti-Mouse-IgG-H-L-Highly-Cross-Adsorbed-Secondary-Antibody-Polyclonal/A10037 |
| **donkey anti-rabbit IgG Alexa Fluor 488** | Invitrogen | A32790 | 1:500 (IF) | https://www.thermofisher.com/antibody/product/Donkey-anti-Rabbit-IgG-H-L-Highly-Cross-Adsorbed-Secondary-Antibody-Polyclonal/A32790 |
| **donkey anti-rat IgG Alexa Fluor 647** | Invitrogen | A48272 | 1:500 (IF) | https://www.thermofisher.com/antibody/product/Donkey-anti-Rat-IgG-H-L-Highly-Cross-Adsorbed-Secondary-Antibody-Polyclonal/A48272 |
| **GATA6** | R&D Systems | AF1700 | 1:50 (IF) | https://www.rndsystems.com/products/human-gata-6-antibody_af1700 |
| **H3K27ac** | GeneTex | GTX128944 | 2 µg (ChIP) | https://www.genetex.com/Product/Detail/Histone-H3K27ac-Acetyl-Lys27-antibody/GTX128944 |
| **HA-Tag** | Cell Signaling Technology | 3724 | 1:1000 (IHC) | https://www.cellsignal.com/products/primary-antibodies/ha-tag-c29f4-rabbit-mab/3724?_requestid=575450 |
| **HDAC1** | Millipore | 06-720 | 1:100 (WB), 5 µg (ChIP) | https://www.merckmillipore.com/DE/de/product/Anti-HDAC1-Antibody,MM_NF-06-720 |
| **HRP anti-goat IgG** | Santa Cruz | sc-2020 | 1:5000 (WB) | https://datasheets.scbt.com/sc-2020.pdf |
| **HRP anti-mouse IgG** | Cell Signaling Technology | 7076S | 1:6000 (WB) | https://www.cellsignal.de/products/secondary-antibodies/anti-mouse-igg-hrp-linked-antibody/7076?site-search-type=Products&N=4294956287&Ntt=7076s&fromPage=plp&_requestid=2251506 |

| | | | | |
|---|---|---|---|---|
| **HRP anti-rabbit IgG** | Cell Signaling Technology | 7074S | 1:6000 (WB) | https://www.cellsignal.de/products/secondary-antibodies/anti-rabbit-igg-hrp-linked-antibody/7074?site-search-type=Products&N=4294956287&Ntt=7074s&fromPage=plp&_requestid=2251522 |
| **HRP β-actin** | Sigma-Aldrich | A3854 | 1:40000 (WB) | https://www.sigmaaldrich.com/DE/en/product/sigma/a3854?context=product |
| **JUNB** | Cell Signaling Technology | 3753 | 1:100 (IF), 1:300 (IHC), 1:1000 (WB), 5 µg (co-IP), 5 µg (ChIP) | https://www.cellsignal.de/products/primary-antibodies/junb-c37f9-rabbit-mab/3753?site-search-type=Products&N=4294956287&Ntt=3753s&fromPage=plp&_requestid=2228795 |
| **mouse anti-rabbit IgG (light-chain-specific)** | Cell Signaling Technology | 93702 | 1:2000 (WB) | https://www.cellsignal.com/products/secondary-antibodies/mouse-anti-rabbit-igg-light-chain-specific-d4w3e-mab-hrp-conjugate/93702?_requestid=475656 |
| **normal rabbit IgG** | Millipore | 12-370 | 1.5 µg (co-IP) | https://www.merckmillipore.com/DE/de/product/Normal-Rabbit-IgG,MM_NF-12-370 |
| **pan-cytokeratin** | Santa Cruz | sc-81714 | 1:100 (IF) | https://www.scbt.com/de/p/pan-cytokeratin-antibody-ae1-ae3 |
| **rabbit IgG** | Diagenode | C15410206 | 2 µg (ChIP) | https://www.diagenode.com/en/p/rabbit-igg-250-ug-250-ul |
| **TNF-α** | Abcam | ab1793 | 1:50 (IF), 1:400 (IHC) | https://www.abcam.com/tnf-alpha-antibody-52b83-ab1793.html |

WB, Western blot
IF, Immunofluorescence
IHC, Immunohistochemistry
FC, flow cytometry
co-IP, co-immunoprecipitation
ChIP, chromatin immunoprecipitation

## Supplementary Table 2. Primer sequences and siRNAs

| Gene | Sequence/Assay ID | Application | Species | Company/Source |
|---|---|---|---|---|
| **JUNB F** | GGGCTCTGGGTTCCCTCATA | ChIP-qPCR | Human | Sigma Aldrich |
| **JUNB R** | ATCTGAGAGCTTCCCTCCCC | ChIP-qPCR | Human | Sigma Aldrich |
| **HNF1B F** | AACCCCAAGTGGATGCTTACC | ChIP-qPCR | Human | Sigma Aldrich |
| **HNF1B R** | TTGTGTCAACCCATGGGGAC | ChIP-qPCR | Human | Sigma Aldrich |
| **FOXA1 F** | GACGACCGTCTGGTTTCTGAT | ChIP-qPCR | Human | Sigma Aldrich |
| **FOXA1 R** | CCTTGCTCTCCCAGCTAACG | ChIP-qPCR | Human | Sigma Aldrich |
| **Silencer Negative Control No.1 siRNA** | AM4635 | siRNA | Human | Ambion |
| **JUNB** | AM16708 | siRNA | Human | Ambion |
| **HDAC1** | SASI_Hs01_00079968 | siRNA | Human | Sigma Aldrich |
| **JUNB F** | AACAGCCCTTCTACCACGAC | RT-qPCR | Human | Sigma Aldrich |
| **JUNB R** | CAGGCTCGGTTTCAGGAGTT | RT-qPCR | Human | Sigma Aldrich |
| **cJUN F** | TCCAAGTGCCGAAAAAGGAAG | RT-qPCR | Human | Sigma Aldrich |
| **cJUN R** | CGAGTTCTGAGCTTTCAAGGT | RT-qPCR | Human | Sigma Aldrich |
| **IL1A F** | GTAGCCACGCCTACTTAAGAC | RT-qPCR | Human | Sigma Aldrich |
| **IL1A R** | ACATGTCTGGAACTTTGGCC | RT-qPCR | Human | Sigma Aldrich |
| **IL1B F** | ATGCACCTGTACGATCACTG | RT-qPCR | Human | Sigma Aldrich |
| **IL1B R** | ACAAAGGACATGGAGAACACC | RT-qPCR | Human | Sigma Aldrich |
| **CXCL10 F** | GAAAGCAGTTAGCAAGGAAAGGT | RT-qPCR | Human | Sigma Aldrich |
| **CXCL10 R** | ATGTAGGGAAGTGATGGGAGAGG | RT-qPCR | Human | Sigma Aldrich |
| **CCL2 F** | CAGCCAGATGCAATCAATGCC | RT-qPCR | Human | Sigma Aldrich |
| **CCL2 R** | TGGAATCCTGAACCCACTTCT | RT-qPCR | Human | Sigma Aldrich |
| **cJUN F** | ATGCCATCAGCATATGCCCTTA | ChIP-qPCR | Human | Sigma Aldrich |
| **cJUN R** | GCAGAGGTGCTGCCTCTTAG | ChIP-qPCR | Human | Sigma Aldrich |
| **IL1A F** | TAGATGAGGTGTTGCGTGTCTTG | ChIP-qPCR | Human | Sigma Aldrich |
| **IL1A R** | GGCAAGTTTATGTGACACAGGC | ChIP-qPCR | Human | Sigma Aldrich |
| **IL1B F** | ACAAACAGAAATTTGGGGTGTGC | ChIP-qPCR | Human | Sigma Aldrich |
| **IL1B R** | AGACTTGCCGAGGTCCTTCT | ChIP-qPCR | Human | Sigma Aldrich |
| **CXCL9/10 F** | CAGAACCCCTTACCTTGCACTC | ChIP-qPCR | Human | Sigma Aldrich |
| **CXCL9/10 R** | AAAAGTGGTGTTCTTTTCCTCTTGG | ChIP-qPCR | Human | Sigma Aldrich |
| **GATA6 F** | GTCCCATCCAACAGAACGGG | ChIP-qPCR | Human | Sigma Aldrich |
| **GATA6 R** | AAAGGGAAGCACTGTTGATTCCTA | ChIP-qPCR | Human | Sigma Aldrich |
| **XS13 F** | TGGGACAGAACACCATGATG | RT-PCR | Human | Sigma Aldrich |
| **XS13 R** | AGTTTCTCCAGAGCTGGGTTGT | RT-PCR | Human | Sigma Aldrich |

F, forward
R, reverse

## Supplementary Table 3. Directly JUNB-repressed Genes in CAPAN1

| Gene Symbol | log2FoldChange (in siJUNB RNA-seq) | *P*adj (in siJUNB RNA-seq) |
|---|---|---|
| CD9 | 2.30302714 | 7.33207E-11 |
| BHLHA15 | 3.180207177 | 5.99872E-10 |
| IL6ST | 2.747851072 | 9.45274E-09 |
| HIF1A | 2.447740877 | 3.02188E-08 |
| PLP2 | 1.80176869 | 3.09147E-07 |
| MAFK | 3.223784012 | 4.19066E-07 |
| PLAT | 1.678812456 | 1.2072E-06 |
| NTN4 | 2.383368699 | 2.05437E-06 |
| ANXA4 | 1.634604525 | 2.87598E-06 |
| ATP1A1 | 2.231312923 | 3.01269E-06 |
| DDR1 | 3.410001912 | 4.3401E-06 |
| PPP1CB | 1.518328353 | 5.19404E-06 |
| ARF6 | 1.585621283 | 8.41588E-06 |
| TINAGL1 | 1.433384631 | 1.23122E-05 |
| CHML | 2.232883726 | 1.24132E-05 |
| PPL | 1.783821822 | 2.24891E-05 |
| TNFAIP2 | 1.486074633 | 2.24891E-05 |
| MAP3K1 | 2.276656941 | 2.42692E-05 |
| JUN | 1.660409566 | 2.45928E-05 |
| CLDN1 | 1.46666729 | 2.58066E-05 |
| KTN1 | 1.566163247 | 2.60416E-05 |
| ANO1 | 1.588631642 | 2.8937E-05 |
| F3 | 1.312868162 | 2.99466E-05 |
| SGK1 | 1.80460572 | 3.41108E-05 |
| MYC | 1.495161179 | 3.65332E-05 |
| DDIT4 | 2.269014187 | 5.11048E-05 |
| GRHL3 | 3.627397554 | 5.58288E-05 |
| OXR1 | 2.455916214 | 6.04852E-05 |
| CCNL1 | 1.500127328 | 8.51445E-05 |
| SPRED2 | 2.230822497 | 8.54011E-05 |
| LPIN2 | 1.888005533 | 9.47938E-05 |
| RHOB | 1.656557994 | 9.89007E-05 |
| HERC1 | 1.991552052 | 0.00011371 |
| PHLDB2 | 2.097270643 | 0.000119872 |
| TOB1 | 2.176445404 | 0.000121393 |
| TIPARP | 1.825791704 | 0.000136934 |
| KCTD18 | 2.807170977 | 0.000147421 |
| LAMA3 | 1.993787268 | 0.000161416 |
| CAND1 | 1.333303138 | 0.000174336 |
| SLC25A25 | 2.33912962 | 0.000175331 |
| NCEH1 | 1.678009346 | 0.000225594 |
| FTH1 | 1.131267619 | 0.000279524 |
| ATP13A3 | 1.722355476 | 0.000302585 |

| | | |
|---|---|---|
| ZFP36L2 | 3.932226594 | 0.000311096 |
| TRIM31 | 3.145761212 | 0.000340758 |
| F2RL1 | 1.822406801 | 0.000373476 |
| CDH1 | 1.279637258 | 0.000419781 |
| KRR1 | 1.379950931 | 0.000480475 |
| RIPK4 | 1.805202792 | 0.000480475 |
| RNF19A | 1.64587953 | 0.000490347 |
| PLEKHA7 | 1.660460041 | 0.000562665 |
| ZFAND5 | 1.256556255 | 0.000595708 |
| DAGLA | 3.36933566 | 0.000656819 |
| CAB39 | 1.226549688 | 0.000665577 |
| EFNB2 | 1.208674026 | 0.000681357 |
| PLK2 | 1.087557909 | 0.000698044 |
| NFKBIZ | 1.673241242 | 0.000781286 |
| TMEM87B | 1.458084408 | 0.000814036 |
| PDGFB | 1.499169985 | 0.000862807 |
| EHF | 1.234688986 | 0.000883477 |
| SMNDC1 | 1.698117527 | 0.000902329 |
| TGIF1 | 1.358178158 | 0.001027115 |
| RAI1 | 1.866993338 | 0.001125674 |
| RPRD1B | 1.404003744 | 0.001135337 |
| TNS3 | 1.389959694 | 0.001271812 |
| MYH9 | 1.21054785 | 0.001420118 |
| GLS | 1.554649719 | 0.001472254 |
| BZW1 | 1.215038193 | 0.001478993 |
| ZNF217 | 1.887834925 | 0.001661629 |
| SLC30A1 | 1.577228903 | 0.001832954 |
| MYD88 | 1.327506312 | 0.002112998 |
| MTUS1 | 2.030789989 | 0.002178007 |
| EPHA2 | 1.077483431 | 0.002315668 |
| DUSP5 | 1.210022532 | 0.002349691 |
| TAF1B | 2.325783757 | 0.002416566 |
| EPCAM | 0.970649333 | 0.002424424 |
| ARFGAP3 | 1.318657887 | 0.002557899 |
| MPZL2 | 1.133568311 | 0.002899863 |
| ASAP1 | 1.504804894 | 0.003152605 |
| RREB1 | 1.569039704 | 0.0032775 |
| TRIO | 1.348058973 | 0.003797991 |
| MAML2 | 1.729793758 | 0.003797991 |
| ZNF710 | 2.085509564 | 0.00415095 |
| SH3GLB1 | 0.996046914 | 0.004310042 |
| SOX9 | 1.23548912 | 0.004703012 |
| MBNL2 | 1.238299692 | 0.004732936 |
| OPA1 | 1.121666081 | 0.005214639 |
| IRF2BP2 | 1.191725561 | 0.005363801 |
| IL1R1 | 1.966296789 | 0.005450073 |
| MSX2 | 1.657135135 | 0.00566226 |
| CLIC5 | 1.355591379 | 0.005687845 |

| | | |
|---|---|---|
| SMAD3 | 0.917370556 | 0.005995235 |
| CDC42EP3 | 1.070811091 | 0.006230274 |
| FAM131A | 2.019200017 | 0.006279655 |
| OSBPL3 | 0.981981729 | 0.00647958 |
| RBBP8 | 1.113692962 | 0.007137216 |
| SPEN | 1.291133703 | 0.007706401 |
| GPR176 | 1.454121209 | 0.008747785 |
| ERRFI1 | 0.933999607 | 0.00884348 |
| NEDD9 | 0.937068812 | 0.009050678 |
| YWHAQ | 0.987784133 | 0.009062016 |
| SLMAP | 1.047096766 | 0.009301304 |
| AHR | 1.26967732 | 0.009421802 |
| ADAM10 | 0.893139456 | 0.009713759 |
| SNX27 | 1.302256375 | 0.009883648 |
| GCH1 | 1.49696263 | 0.010558729 |
| VEGFA | 1.169346461 | 0.011353118 |
| MYO1E | 1.026381615 | 0.011353118 |
| CD55 | 0.979841932 | 0.012670145 |
| PARD6B | 1.553378542 | 0.014319636 |
| CSRNP1 | 1.305563804 | 0.014670521 |
| KLK6 | 1.072626098 | 0.014741315 |
| ANKRD46 | 1.753324066 | 0.014960387 |
| PDE8A | 1.063387264 | 0.014997496 |
| GET4 | 1.488930419 | 0.01578336 |
| ARHGAP23 | 1.034670828 | 0.016560822 |
| AFAP1L2 | 1.256413651 | 0.016939135 |
| DUSP10 | 1.221907707 | 0.017606465 |
| THBS1 | 1.268405353 | 0.017794312 |
| YWHAZ | 0.772357947 | 0.017948276 |
| ERMP1 | 1.094534226 | 0.019715558 |
| SGPL1 | 0.949456009 | 0.020737023 |
| PPP1R18 | 5.919115664 | 0.020969224 |
| GDA | 0.75116901 | 0.021441724 |
| MAP3K5 | 1.223516381 | 0.021724458 |
| IFT20 | 1.179595389 | 0.022553009 |
| EPAS1 | 1.051162154 | 0.023503222 |
| CAPZA2 | 0.970699922 | 0.024654616 |
| SPRY4 | 1.48222772 | 0.025539131 |
| SMAD6 | 1.155673718 | 0.028782632 |
| EML2 | 0.941757234 | 0.029383165 |
| PTPN1 | 0.952297582 | 0.029477217 |
| PRKCH | 1.01084251 | 0.029851731 |
| DUSP1 | 1.787434708 | 0.030084226 |
| HES1 | 1.09108248 | 0.03263226 |
| SYTL2 | 1.381284947 | 0.03314078 |
| IRF2BPL | 1.236709393 | 0.033709639 |
| GNA13 | 1.262670634 | 0.035080394 |
| XXYLT1 | 1.100653976 | 0.035484566 |

| | | |
|---|---|---|
| SGMS1 | 1.791918421 | 0.03550677 |
| SLC23A2 | 1.114175652 | 0.037763564 |
| C2CD3 | 1.165396244 | 0.038041572 |
| IGFL1 | 2.159619764 | 0.038210759 |
| TP63 | 5.221382537 | 0.041927798 |
| FOXQ1 | 0.876099828 | 0.047890653 |
| ESYT2 | 0.80374286 | 0.049447904 |

Log2FoldChange and *P*adj determined using DESeq2. *P*adj refers to Wald test *P* value adjusted by Benjamini and Hochberg method.

## Supplementary Table 4. JUNB repression signature

| Gene Symbol |
|---|
| OPA1 |
| SGMS1 |
| XXYLT1 |
| PLK2 |
| PHLDB2 |
| FAM131A |
| PPP1CB |
| YWHAQ |
| IFT20 |
| GNA13 |
| TAF1B |
| CAPZA2 |
| RBBP8 |
| NCEH1 |
| C2CD3 |
| CDC42EP3 |
| TNS3 |
| MYH9 |
| OSBPL3 |
| YWHAZ |
| CHML |
| ANO1 |
| THBS1 |
| SGPL1 |
| MBNL2 |
| RPRD1B |
| SOX9 |
| OXR1 |
| PTPN1 |
| HIF1A |
| RNF19A |
| SMNDC1 |
| SLC30A1 |
| MSX2 |
| TMEM87B |
| ANKRD46 |
| EFNB2 |
