# Supplementary material for: Spatial tumor immune heterogeneity facilitates subtype co-existence and therapy response in pancreatic cancer
Source: Nat Commun. 2025 Jan 6;16:335. doi: 10.1038/s41467-024-55330-7 (PMC11704331; doi:10.1038/s41467-024-55330-7)
Supplement: Supplementary file 3 — Reporting Summary [file 41467_2024_55330_MOESM3_ESM.pdf]

nature portfolio

Corresponding author(s): Shiv K. Singh

Last updated by author(s): Nov 29, 2024

Reporting Summary

Nature Portfolio wishes to improve the reproducibility of the work that we publish. This form provides structure for consistency and transparency in reporting. For further information on Nature Portfolio policies, see our Editorial Policies and the Editorial Policy Checklist.

Statistics

For all statistical analyses, confirm that the following items are present in the figure legend, table legend, main text, or Methods section.

n/a	Confirmed	
☐	☒	The exact sample size (*n*) for each experimental group/condition, given as a discrete number and unit of measurement
☐	☒	A statement on whether measurements were taken from distinct samples or whether the same sample was measured repeatedly
☐	☒	The statistical test(s) used AND whether they are one- or two-sided<br>*Only common tests should be described solely by name; describe more complex techniques in the Methods section.*
☒	☐	A description of all covariates tested
☐	☒	A description of any assumptions or corrections, such as tests of normality and adjustment for multiple comparisons
☐	☒	A full description of the statistical parameters including central tendency (e.g. means) or other basic estimates (e.g. regression coefficient) AND variation (e.g. standard deviation) or associated estimates of uncertainty (e.g. confidence intervals)
☐	☒	For null hypothesis testing, the test statistic (e.g. *F*, *t*, *r*) with confidence intervals, effect sizes, degrees of freedom and *P* value noted<br>*Give P values as exact values whenever suitable.*
☒	☐	For Bayesian analysis, information on the choice of priors and Markov chain Monte Carlo settings
☒	☐	For hierarchical and complex designs, identification of the appropriate level for tests and full reporting of outcomes
☐	☒	Estimates of effect sizes (e.g. Cohen's *d*, Pearson's *r*), indicating how they were calculated

Our web collection on statistics for biologists contains articles on many of the points above.

Software and code

Policy information about availability of computer code

Data collection	qRT-PCR data was collected with StepOne Plus Software (Applied Biosystems).<br>Western blot/immune blot images were aquired using ChemoStar Software (Intas).<br>Luciferase reporter assay was analyzed using LUmo microplate reader (Autobio Diagnostics).<br>Immunofluorescence images were acquired using FV10-ASW Version 4.2 Software (Olympus).<br>Images of hybridized microarrays were acquired with a DNA microarray scanner (Agilent G2505B).<br>Tile image scans were aquired using VS120 virtual slide microscope and cellSens Dimension software (Olympus).

Data analysis	qRT-PCR data analyzed with StepOne Plus Software v2.3 (Applied Biosystems).<br>For RNA-seq: FastQC v0.11.5, STAR v2.5.2b and Galaxy version v2.7.5b, HTSeq-count version 0.10.0 and Galaxy version 0.9.1 , DESeq2 version 1.22.2, R version 3.5.2, ggplot2 version 3.1.0, pheatmap version 1.0.12 and clusterProfiler version 3.10.1 R packages.<br>RNA-seq in tumor tissues: STAR v2.7.3a, XenofilteR package v1.6 in R v4.1.0 and MCPcounter package v1.2.0 in R v4.2.0 were used for deconvolution and relative abundance of human (tumor cell) and murine (host stroma) compartments.<br>Meta-pathway analysis (Metascape; https://metascape.org/) was used for the annotation of JUNB-bound and ATAC-seq peak regions.<br>R package rGREAT61 v3.0.0 was used for integration of RNA- and ChIP-seq data.<br>For Immunofluorescence images, QuPath v0.4.3, ImageJ Fiji Software version 1.50b and cellSens Dimensions Desktop 2.3 (Olympus).<br>For patient survival analysis the R2 platform (R2: Genomics Analysis and Visualization Platform (http://r2.amc.nl)). For compartment-specific patient RNA-seq: R package MCP counter v1.1 and clusterProfiler version 3.14.3.<br>CIBERSORTx analysis (https://cibersortx.stanford.edu) and LM22 signature matrix.Expression heatmaps by the pheatmap package v1.0.12.<br>For FACS, BD FACSDIVA Software and Flowjo V10.1. Statistical analysis was performed using GraphPad Prism version 8.0.2.<br>Patient survival analysis (v3.5-5) and (ggsurvfit v0.3.0) packages.

For manuscripts utilizing custom algorithms or software that are central to the research but not yet described in published literature, software must be made available to editors and reviewers. We strongly encourage code deposition in a community repository (e.g. GitHub). See the Nature Portfolio guidelines for submitting code & software for further information.

Data

Policy information about availability of data

All manuscripts must include a data availability statement. This statement should provide the following information, where applicable:

- Accession codes, unique identifiers, or web links for publicly available datasets
- A description of any restrictions on data availability
- For clinical datasets or third party data, please ensure that the statement adheres to our policy

For this study, the Molecular Signatures Database (https://www.gsea-msigdb.org/gsea/msigdb/) database was used. Previously published21,25 ChIP- and ATAC-seq data are available at Gene Expression Omnibus (GEO) under accession codes GSE173159 [https://www.ncbi.nlm.nih.gov/geo/query/acc.cgi?acc=GSE173159] and GSE64560 [https://www.ncbi.nlm.nih.gov/geo/query/acc.cgi?acc=GSE64560]. ChIP- and RNA-seq data generated in this study has been deposited at GEO with the accession code GSE276324 [https://www.ncbi.nlm.nih.gov/geo/query/acc.cgi?acc=GSE276324]. FACS-sorted epithelial patient tumor RNA-seq data22 are available at EGA under accession code EGAS00001004660 [https://ega-archive.org/studies/EGAS00001004660]. Access is restricted due to patient data protection but can be made available through the DKFZ-HIPO Data Access Committee of Heidelberg Center for Personalized Oncology (hipo_daco@dkfz-heidelberg.de) using the Data Access Request via the EGA DAC Portal. Patient tumor LCM-enriched transcriptome and proteome data are available at EGA under accession code EGAS00001002543 [https://ega-archive.org/studies/EGAS00001002543] and from UCSD's MASSive database under accession code MSV000086812 [https://massive.ucsd.edu/ProteoSAFe/dataset.jsp?task=35d2ed0cbcd04045adceeb4866e478a3]. Data access can be requested from the PanCuRx Translational Research Initiative using the OICR Data Access Agreement as detailed on EGA. Processed proteome data is detailed in the previous publication43 Table S7 [https://ars.els-cdn.com/content/image/1-s2.0-S0092867421011053-mmc6.xlsx].
Source data are provided with this paper.

Research involving human participants, their data, or biological material

Policy information about studies with human participants or human data. See also policy information about sex, gender (identity/presentation), and sexual orientation and race, ethnicity and racism.

Reporting on sex and gender	PDAC resection tissue utilized for IF staining of the UMG cohort included 32 chemotherapy-naïve, resected primary PDAC cases, with 23 female and 9 male patients, 5 stage I, 19 stage II, and 8 stage III, with a mean age at diagnosis of 72.2 years (age range 49-86 years).<br>The PDAC tissue microarray (TMA) cohort from PMCC comprises resectable primary pancreatic tumor specimens from 105 treatment-naïve patients diagnosed with PDAC. These included 50 female and 65 male patients, 9 stage I and 96 stage II, with a mean age at diagnosis of 65.8 years (age range 42-84 years).<br>Data from 31 primary resected PDACs have been used in this study. These comprise 16 tumors derived from female patients and 13 from male patients. The average age of the patients was 66 years old (ranging from 41 to 83 years old).
Reporting on race, ethnicity, or other socially relevant groupings	Race, ethnicity, and other socially relevant groupings were not collected in this study. They were not relevant for the outcome of this study.
Population characteristics	not applicable
Recruitment	Tissue samples were collected from patients who underwent partial pancreatoduodenectomy at the Department of General, Visceral, and Transplantation Surgery, University of Heidelberg, without self-selection or other biases.
Ethics oversight	The study included 31 patients, out of which 16 were females and 13 were males. All patients were part of the HIPO-project and the study was approved by the ethical committee of the University of Heidelberg, under case number S-206/2011 and EPZ-Biobank Ethic Vote #301/2001. The study was conducted in accordance with the Helsinki Declaration and written informed consent was obtained from all patients.<br>PDAC resection tissue utilized for IF staining was derived from the Molecular Pancreatic Cancer Program (MolPAC) of the University Medical Center Göttingen as part of the study "Klinische und molekulare Evaluation von Patienten mit Pankreasraumforderungen im Rahmen des Pankreasprogramms der UMG (MolPAC)" (ethics approval 11/5/17).<br>PDAC patients tumors were obtained from the UHN Biospecimens Program after collection at Princess Margaret Cancer Centre (PMCC; Toronto, Canada) and have been previously discussed in studies (Connor et al., 2019; Grünwald et al, 2021, Aliar et al., 2023). All patients provided written informed consent for the molecular characterization of their tumor samples

and for follow-up on their clinical information and were approved by University Health Network Research Ethics Board (case numbers 03-0049, 08-0767, 15-9596, 17-6106, 16-5380).

Note that full information on the approval of the study protocol must also be provided in the manuscript.

Field-specific reporting

Please select the one below that is the best fit for your research. If you are not sure, read the appropriate sections before making your selection.

☒ Life sciences ☐ Behavioural & social sciences ☐ Ecological, evolutionary & environmental sciences

For a reference copy of the document with all sections, see nature.com/documents/nr-reporting-summary-flat.pdf

Life sciences study design

All studies must disclose on these points even when the disclosure is negative.

Sample size	The sample sizes used in our experiments were determined based on our previous experimental experience, as described in Nature Cancer 2021 (PMID: 35122059) and JCI Insight 2022 (PMID: 35993361). The figure legends provide details about the sample size, number of replicates, and statistical analyses used for each experiment. All experiments included replicates and were analyzed at least two to three times, unless otherwise stated in the manuscript. For orthotopic xenograft experiments, we used more than three mice per group, as mentioned in the figure legends for each experiment.
Data exclusions	For sequencing analyses, samples were removed if they did not pass overall quality control assessment. No other data was excluded from the analysis.
Replication	Multiple repeats were performed to generate reproducible results, as described in the methods, figure legends, and extended figure legends.
Randomization	All in vitro samples and animals were randomized according to the instructions provided in the text, methods, figures, and figure legends.
Blinding	Two independent researchers conducted various methods such as 3D invasion assay, Reporter assay, qPCR, ChIP, immunoblot, and library preparations. Blinding was not applicable for the measurement of tumor growth through ultrasonography or treatment conditions for animal experiments. However, histopathological characterization of tumor-bearing mice and flow cytometry were performed in a blinded fashion.

Behavioural & social sciences study design

All studies must disclose on these points even when the disclosure is negative.

Study description	*Briefly describe the study type including whether data are quantitative, qualitative, or mixed-methods (e.g. qualitative cross-sectional, quantitative experimental, mixed-methods case study).*
Research sample	*State the research sample (e.g. Harvard university undergraduates, villagers in rural India) and provide relevant demographic information (e.g. age, sex) and indicate whether the sample is representative. Provide a rationale for the study sample chosen. For studies involving existing datasets, please describe the dataset and source.*
Sampling strategy	*Describe the sampling procedure (e.g. random, snowball, stratified, convenience). Describe the statistical methods that were used to predetermine sample size OR if no sample-size calculation was performed, describe how sample sizes were chosen and provide a rationale for why these sample sizes are sufficient. For qualitative data, please indicate whether data saturation was considered, and what criteria were used to decide that no further sampling was needed.*
Data collection	*Provide details about the data collection procedure, including the instruments or devices used to record the data (e.g. pen and paper, computer, eye tracker, video or audio equipment) whether anyone was present besides the participant(s) and the researcher, and whether the researcher was blind to experimental condition and/or the study hypothesis during data collection.*
Timing	*Indicate the start and stop dates of data collection. If there is a gap between collection periods, state the dates for each sample cohort.*
Data exclusions	*If no data were excluded from the analyses, state so OR if data were excluded, provide the exact number of exclusions and the rationale behind them, indicating whether exclusion criteria were pre-established.*
Non-participation	*State how many participants dropped out/declined participation and the reason(s) given OR provide response rate OR state that no participants dropped out/declined participation.*
Randomization	*If participants were not allocated into experimental groups, state so OR describe how participants were allocated to groups, and if allocation was not random, describe how covariates were controlled.*

Ecological, evolutionary & environmental sciences study design

All studies must disclose on these points even when the disclosure is negative.

Study description	*Briefly describe the study. For quantitative data include treatment factors and interactions, design structure (e.g. factorial, nested, hierarchical), nature and number of experimental units and replicates.*
Research sample	*Describe the research sample (e.g. a group of tagged Passer domesticus, all Stenocereus thurberi within Organ Pipe Cactus National Monument), and provide a rationale for the sample choice. When relevant, describe the organism taxa, source, sex, age range and any manipulations. State what population the sample is meant to represent when applicable. For studies involving existing datasets, describe the data and its source.*
Sampling strategy	*Note the sampling procedure. Describe the statistical methods that were used to predetermine sample size OR if no sample-size calculation was performed, describe how sample sizes were chosen and provide a rationale for why these sample sizes are sufficient.*
Data collection	*Describe the data collection procedure, including who recorded the data and how.*
Timing and spatial scale	*Indicate the start and stop dates of data collection, noting the frequency and periodicity of sampling and providing a rationale for these choices. If there is a gap between collection periods, state the dates for each sample cohort. Specify the spatial scale from which the data are taken*
Data exclusions	*If no data were excluded from the analyses, state so OR if data were excluded, describe the exclusions and the rationale behind them, indicating whether exclusion criteria were pre-established.*
Reproducibility	*Describe the measures taken to verify the reproducibility of experimental findings. For each experiment, note whether any attempts to repeat the experiment failed OR state that all attempts to repeat the experiment were successful.*
Randomization	*Describe how samples/organisms/participants were allocated into groups. If allocation was not random, describe how covariates were controlled. If this is not relevant to your study, explain why.*
Blinding	*Describe the extent of blinding used during data acquisition and analysis. If blinding was not possible, describe why OR explain why blinding was not relevant to your study.*

Did the study involve field work? ☐ Yes ☒ No

Field work, collection and transport

Field conditions	*Describe the study conditions for field work, providing relevant parameters (e.g. temperature, rainfall).*
Location	*State the location of the sampling or experiment, providing relevant parameters (e.g. latitude and longitude, elevation, water depth).*
Access & import/export	*Describe the efforts you have made to access habitats and to collect and import/export your samples in a responsible manner and in compliance with local, national and international laws, noting any permits that were obtained (give the name of the issuing authority, the date of issue, and any identifying information).*
Disturbance	*Describe any disturbance caused by the study and how it was minimized.*

Reporting for specific materials, systems and methods

We require information from authors about some types of materials, experimental systems and methods used in many studies. Here, indicate whether each material, system or method listed is relevant to your study. If you are not sure if a list item applies to your research, read the appropriate section before selecting a response.

Materials & experimental systems

n/a	Involved in the study	
☐	☒	Antibodies
☐	☒	Eukaryotic cell lines
☒	☐	Palaeontology and archaeology
☐	☒	Animals and other organisms
☒	☐	Clinical data
☒	☐	Dual use research of concern
☒	☐	Plants

Methods

n/a	Involved in the study	
☐	☒	ChIP-seq
☐	☒	Flow cytometry
☒	☐	MRI-based neuroimaging

Antibodies

Antibodies used	Name; Company; Catalog No.; Application and Dilution<br>anti-CD3 antibody; abcam; ab16669;1:200 (IHC)<br>anti-CD8 antibody; abcam; ab217344;1:1000 (IHC)<br>anti-CD31; Miltenyi Biotec; REA730; 1:50 (FC)<br>anti-CD326 (EpCAM); Miltenyi Biotec; clone HEA-125;1:10 (FC)<br>anti-pan-cytokeratin antibody; Santa Cruz; sc-81714; 1:100 (IF)<br>anti-TNFα antibody; abcam; ab1793; 1:50 (IF)<br>anti-GATA6 antibody; R&D Systems; AF1700; 1:50 (IF)<br>anti-CD45 antibody;BD Pharmingen;550539;1:50 (IF)<br>anti-CD45; Miltenyi Biotec; 130-108-020; 1:10 (FC)<br>anti-CD31; Miltenyi Biotec; REA730; 1:50 (FC)<br>anti-HA-Tag antibody; Cell Signaling Technology;3724;1:1000 (IHC)<br>anti-HDAC1 antibody; ;Millipore 06-720;1:100 (WB), 5 µg (ChIP)<br>anti-HDAC1 antibody; Millipore; 06-720;1:100 (WB)<br>anti-HDAC1 antibody; CellSignaling; 2062; 1:1000 (WB)<br>anti-HDAC1 antibody; Active Motif; 40967; 5 µg (ChIP)<br>anti-JunB antibody; CellSignaling; 3753; 1:100 (IF); 1:300 (IHC); 1:1000 (WB); 5µg (Co-IP & ChIP)<br>anti-cJUN antibody; Cell Signaling; 9165S; 1:1000 (WB); 1:100 (IF)<br>anti-CCL2 antibody; Invitrogen; MA5-17040; 1:1000 (WB); 1:500 (IF)<br>anti-CD163 (D6U1) antibody; CellSignaling; 93498; 1:250 (IHC)<br>anti-CD86 (E2G8P) antibody; CellSignaling; 91882; 1:100 (IHC)<br>anti-rabbit IgG;Diagenode;C15410206;2 µg (ChIP)<br>IgG1,k isotype control; BD Biosciences; 553973; 1:20 (FC)<br>anti-CD68 antibody; Abcam; ab955; 1:50 (IF)<br>anti-β-actin antibody; ThermoFisher Scientific; A3854; 1:40000 (WB)<br>anti-H3K27ac antibody; GeneTex; GTX128944; 2 µg per ChIP<br>Rabbit IgG antibody; Diagenode; C15410206; 2 µg per ChIP<br>HRP anti-mouse; Cell Signaling Technology; 7076S; 1:6000 (WB)<br>HRP anti-goat IgG;Santa Cruz;sc-2020;1:5000 (WB)<br>HRP anti-rabbit; Cell Signaling Technology; 7074S; 1:6000 (WB)<br>HRP β-actin;Sigma-Aldrich;A3854;1:40000 (WB)<br>anti-mouse anti-rabbit IgG (light-chain-specific);Cell Signaling Technology;93702;1:2000 (WB)<br>anti-normal rabbit IgG;Millipore;12-370;1.5 µg (co-IP)<br>anti-donkey anti-goat IgG Alexa Fluor 568;Invitrogen;A-11057;1:500 (IF)<br>anti-donkey anti-goat IgG Alexa Fluor 647;Invitrogen;A32849;1:500 (IF)<br>anti-donkey anti-mouse IgG Alexa Fluor 568;Invitrogen; A10037;1:500 (IF)<br>anti-donkey anti-rabbit IgG Alexa Fluor 488;Invitrogen;A32790;1:500 (IF)<br>anti-donkey anti-rat IgG Alexa Fluor 647;Invitrogen;A48272;1:500 (IF)
Validation	The manufacturer's websites were used to validate all antibodies for the applications they were indicated for.

Eukaryotic cell lines

Policy information about cell lines and Sex and Gender in Research

Cell line source(s)	CAPAN1,CFPAC1, HPAFII, MiaPaCa2 and CAPAN2 PDAC cell lines were obtained from ATCC. HEK293T (clone 17) cells were purchased from ATCC . Primary PDAC cell line derived from KPC mice were generated in our department and has been described previously (Patzak et al., 2019). PDX derived cell lines were generated in cooperation with Ruhr University Bochum as described in the methods.
Authentication	CAPAN1, CAPAN2 and MiaPaCa2 cell lines have been verified for their authenticity using RRID:CVCL_0237, RRID:CVCL_0026 and RRID:CVCL_0428 respectively by the German Collection of Microorganisms and cell culture GmbH (DSMZ). CFPAC1 (ATCC CRL-1918; RRID:CVCL_1119) and HPAF-II (ATCC CRL-1997; RRID:CVCL_0313) cell lines were authenticated by the IEO Tissue Culture Facility using the GenePrint10 System (Promega). Additionally, HEK293T (clone 17) cells have also been authenticated by ATCC (CRL-11268). The authentication process involved DNA profiling to amplify short tandem repeat-containing loci.
Mycoplasma contamination	All cell lines tested negative for Mycoplasma under the given conditions.
Commonly misidentified lines<br>(See ICLAC register)	None used.

Palaeontology and Archaeology

Specimen provenance	*Provide provenance information for specimens and describe permits that were obtained for the work (including the name of the issuing authority, the date of issue, and any identifying information). Permits should encompass collection and, where applicable, export.*
Specimen deposition	*Indicate where the specimens have been deposited to permit free access by other researchers.*

Dating methods	*If new dates are provided, describe how they were obtained (e.g. collection, storage, sample pretreatment and measurement), where they were obtained (i.e. lab name), the calibration program and the protocol for quality assurance OR state that no new dates are provided.*

☐ Tick this box to confirm that the raw and calibrated dates are available in the paper or in Supplementary Information.

Ethics oversight	*Identify the organization(s) that approved or provided guidance on the study protocol, OR state that no ethical approval or guidance was required and explain why not.*

Note that full information on the approval of the study protocol must also be provided in the manuscript.

Animals and other research organisms

Policy information about studies involving animals; ARRIVE guidelines recommended for reporting animal research, and Sex and Gender in Research

Laboratory animals	Tumor cell lines were injected orthotopically into 10-week old male NMRI-Foxn1nu/nu and C57Bl/6 mice, while 6-8 week old female NMRI-Foxn1nu/nu mice were used for patient-derived xenografts models. The details regarding the procedures can be found in the text, methods, figures legends and extended data. The animals were kept under 12 hours of daylight and 12 hours of darkness with a temperature of approximately 23°C ± 1°C and humidity between 40-60%.
Wild animals	No wild animal involved in this study.
Reporting on sex	Male NMRI-Foxn1nu/nu and C57Bl/6 mice were used for cell line orthotopic implantation. 6-8 week old female NMRI-Foxn1nu/nu mice were used for patient-derived xenografts models
Field-collected samples	This study did not use any samples collected from the field.
Ethics oversight	All animal experiments were conducted at the UMG following Central Animal-experimental authority guidelines (permission no. 15/2057, 14/1634, 18/2953). The ethics committee of the UMG also approved the generation of the PDX mouse model (permission no. 70112108).

Note that full information on the approval of the study protocol must also be provided in the manuscript.

Clinical data

Policy information about clinical studies

All manuscripts should comply with the ICMJE guidelines for publication of clinical research and a completed CONSORT checklist must be included with all submissions.

Clinical trial registration	*Provide the trial registration number from ClinicalTrials.gov or an equivalent agency.*
Study protocol	*Note where the full trial protocol can be accessed OR if not available, explain why.*
Data collection	*Describe the settings and locales of data collection, noting the time periods of recruitment and data collection.*
Outcomes	*Describe how you pre-defined primary and secondary outcome measures and how you assessed these measures.*

Dual use research of concern

Policy information about dual use research of concern

Hazards

Could the accidental, deliberate or reckless misuse of agents or technologies generated in the work, or the application of information presented in the manuscript, pose a threat to:

No	Yes	
☒	☐	Public health
☒	☐	National security
☒	☐	Crops and/or livestock
☒	☐	Ecosystems
☒	☐	Any other significant area

Experiments of concern

Does the work involve any of these experiments of concern:

No	Yes	
☒	☐	Demonstrate how to render a vaccine ineffective
☒	☐	Confer resistance to therapeutically useful antibiotics or antiviral agents
☒	☐	Enhance the virulence of a pathogen or render a nonpathogen virulent
☒	☐	Increase transmissibility of a pathogen
☒	☐	Alter the host range of a pathogen
☒	☐	Enable evasion of diagnostic/detection modalities
☒	☐	Enable the weaponization of a biological agent or toxin
☒	☐	Any other potentially harmful combination of experiments and agents

Plants

Seed stocks	*Report on the source of all seed stocks or other plant material used. If applicable, state the seed stock centre and catalogue number. If plant specimens were collected from the field, describe the collection location, date and sampling procedures.*
Novel plant genotypes	*Describe the methods by which all novel plant genotypes were produced. This includes those generated by transgenic approaches, gene editing, chemical/radiation-based mutagenesis and hybridization. For transgenic lines, describe the transformation method, the number of independent lines analyzed and the generation upon which experiments were performed. For gene-edited lines, describe the editor used, the endogenous sequence targeted for editing, the targeting guide RNA sequence (if applicable) and how the editor was applied.*
Authentication	*Describe any authentication procedures for each seed stock used or novel genotype generated. Describe any experiments used to assess the effect of a mutation and, where applicable, how potential secondary effects (e.g. second site T-DNA insertions, mosiacism, off-target gene editing) were examined.*

ChIP-seq

Data deposition

☒ Confirm that both raw and final processed data have been deposited in a public database such as GEO.

☒ Confirm that you have deposited or provided access to graph files (e.g. BED files) for the called peaks.

Data access links<br>*May remain private before publication.*	ChIP-seq data are available at Gene Expression Omnibus (GEO) under accession codes GSE173159 [https://www.ncbi.nlm.nih.gov/geo/query/acc.cgi?acc=GSE173159] and under GSE276324 [https://www.ncbi.nlm.nih.gov/geo/query/acc.cgi?acc=GSE276324].
Files in database submission	Please see above link for the details of ChIP-seq files
Genome browser session<br>(e.g. UCSC)	*Provide a link to an anonymized genome browser session for "Initial submission" and "Revised version" documents only, to enable peer review. Write "no longer applicable" for "Final submission" documents.*

Methodology

Replicates	*Describe the experimental replicates, specifying number, type and replicate agreement.*
Sequencing depth	*Describe the sequencing depth for each experiment, providing the total number of reads, uniquely mapped reads, length of reads and whether they were paired- or single-end.*
Antibodies	*Describe the antibodies used for the ChIP-seq experiments; as applicable, provide supplier name, catalog number, clone name, and lot number.*
Peak calling parameters	*Specify the command line program and parameters used for read mapping and peak calling, including the ChIP, control and index files used.*
Data quality	*Describe the methods used to ensure data quality in full detail, including how many peaks are at FDR 5% and above 5-fold enrichment.*
Software	*Describe the software used to collect and analyze the ChIP-seq data. For custom code that has been deposited into a community repository, provide accession details.*

Flow Cytometry

Plots

Confirm that:

☒ The axis labels state the marker and fluorochrome used (e.g. CD4-FITC).

☒ The axis scales are clearly visible. Include numbers along axes only for bottom left plot of group (a 'group' is an analysis of identical markers).

☒ All plots are contour plots with outliers or pseudocolor plots.

☒ A numerical value for number of cells or percentage (with statistics) is provided.

Methodology

Sample preparation	The compartment-sorted patient transcriptome data has been published previously (Espinet et al., Cancer Discovery, 2021). Tumor tissues from sorted human samples were dissociated using the Tumour Dissociating Kit (Milteny Biotec).
Instrument	Sorting was performed via a FACS Fusion system.
Software	The process of collecting data was carried out using BD FACSDIVA software and Flowjo V10.1.
Cell population abundance	The purities after sorting were greater than 95%.
Gating strategy	To isolate the ditinct cell population from a sample, several steps were taken. Firstly, debris was removed by using FSC-H and SSC-H gating. Next, singlets were selected based on FSC-H and FSC-A. Finally, dead cells were excluded using propidium iodide (PI) staining. Epithelial cells were distinguished as EPCAM-FITC+/CD45-VioBlue-. Immune cells were identified as EPCAM-FITC-/CD45-VioBlue+. CAF-enriched cells were defined as EPCAM-FITC-/CD45-VioBlue-, and endothelial cells were labeled as EPCAM-FITC-/CD45-VioBlue-/CD31-APC+

☒ Tick this box to confirm that a figure exemplifying the gating strategy is provided in the Supplementary Information.

Magnetic resonance imaging

Experimental design

Design type	*Indicate task or resting state; event-related or block design.*
Design specifications	*Specify the number of blocks, trials or experimental units per session and/or subject, and specify the length of each trial or block (if trials are blocked) and interval between trials.*
Behavioral performance measures	*State number and/or type of variables recorded (e.g. correct button press, response time) and what statistics were used to establish that the subjects were performing the task as expected (e.g. mean, range, and/or standard deviation across subjects).*

Acquisition

Imaging type(s)	*Specify: functional, structural, diffusion, perfusion.*
Field strength	*Specify in Tesla*
Sequence & imaging parameters	*Specify the pulse sequence type (gradient echo, spin echo, etc.), imaging type (EPI, spiral, etc.), field of view, matrix size, slice thickness, orientation and TE/TR/flip angle.*
Area of acquisition	*State whether a whole brain scan was used OR define the area of acquisition, describing how the region was determined.*

Diffusion MRI ☐ Used ☐ Not used

Preprocessing

Preprocessing software	*Provide detail on software version and revision number and on specific parameters (model/functions, brain extraction, segmentation, smoothing kernel size, etc.).*
Normalization	*If data were normalized/standardized, describe the approach(es): specify linear or non-linear and define image types used for transformation OR indicate that data were not normalized and explain rationale for lack of normalization.*
Normalization template	*Describe the template used for normalization/transformation, specifying subject space or group standardized space (e.g. original Talairach, MNI305, ICBM152) OR indicate that the data were not normalized.*

Noise and artifact removal

Describe your procedure(s) for artifact and structured noise removal, specifying motion parameters, tissue signals and physiological signals (heart rate, respiration).

Volume censoring

Define your software and/or method and criteria for volume censoring, and state the extent of such censoring.

Statistical modeling & inference

Model type and settings

Specify type (mass univariate, multivariate, RSA, predictive, etc.) and describe essential details of the model at the first and second levels (e.g. fixed, random or mixed effects; drift or auto-correlation).

Effect(s) tested

Define precise effect in terms of the task or stimulus conditions instead of psychological concepts and indicate whether ANOVA or factorial designs were used.

Specify type of analysis: ☐ Whole brain ☐ ROI-based ☐ Both

Statistic type for inference

(See Eklund et al. 2016)

Specify voxel-wise or cluster-wise and report all relevant parameters for cluster-wise methods.

Correction

Describe the type of correction and how it is obtained for multiple comparisons (e.g. FWE, FDR, permutation or Monte Carlo).

Models & analysis

n/a	Involved in the study
☐	☐ Functional and/or effective connectivity
☐	☐ Graph analysis
☐	☐ Multivariate modeling or predictive analysis

Functional and/or effective connectivity

Report the measures of dependence used and the model details (e.g. Pearson correlation, partial correlation, mutual information).

Graph analysis

Report the dependent variable and connectivity measure, specifying weighted graph or binarized graph, subject- or group-level, and the global and/or node summaries used (e.g. clustering coefficient, efficiency, etc.).

Multivariate modeling and predictive analysis

Specify independent variables, features extraction and dimension reduction, model, training and evaluation metrics.
