# Supplementary material for: Spatial tumor immune heterogeneity facilitates subtype co-existence and therapy response in pancreatic cancer
Source: Nat Commun. 2025 Jan 6;16:335. doi: 10.1038/s41467-024-55330-7 (PMC11704331; doi:10.1038/s41467-024-55330-7)

Figure 3g

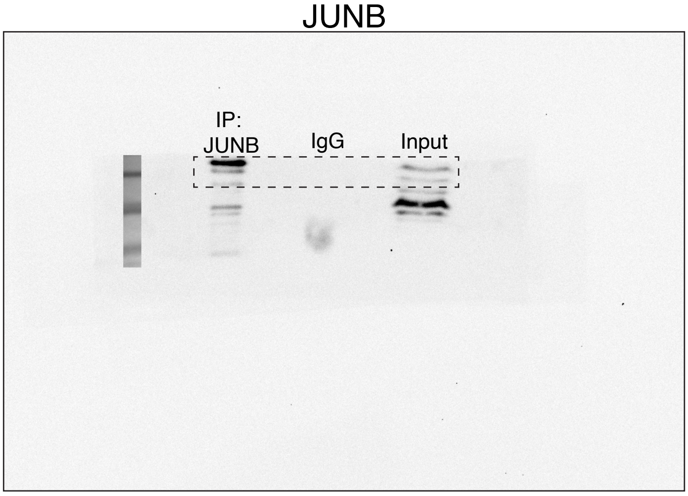
JUNB
IP:
JUNB
IgG
Input

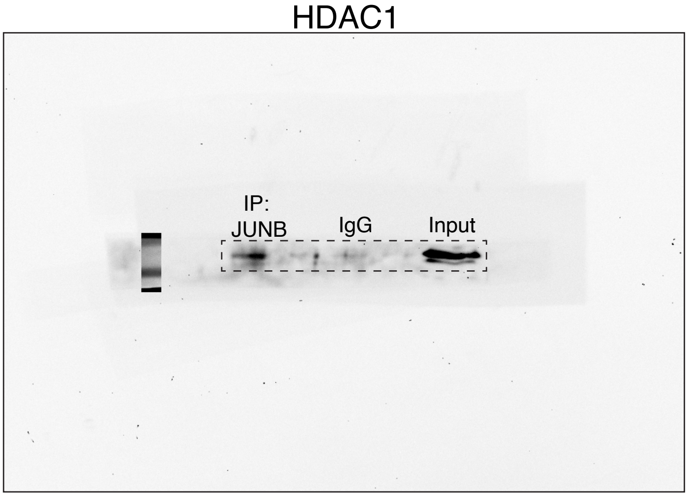
HDAC1
IP:
JUNB
IgG
Input

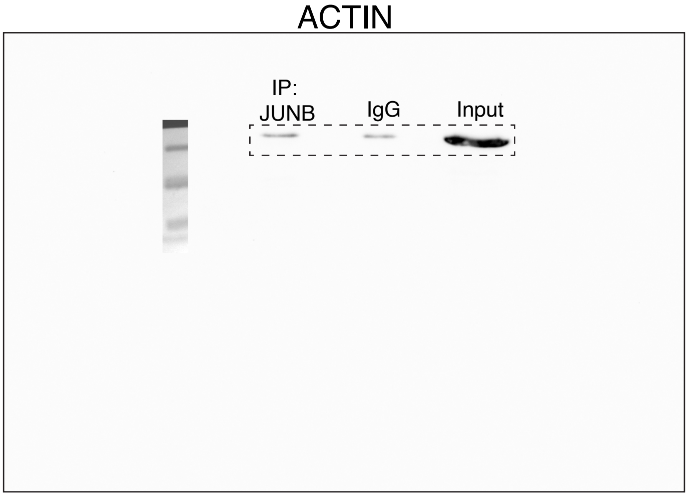
ACTIN
IP:
JUNB
IgG
Input

Figure 3h

JUNB

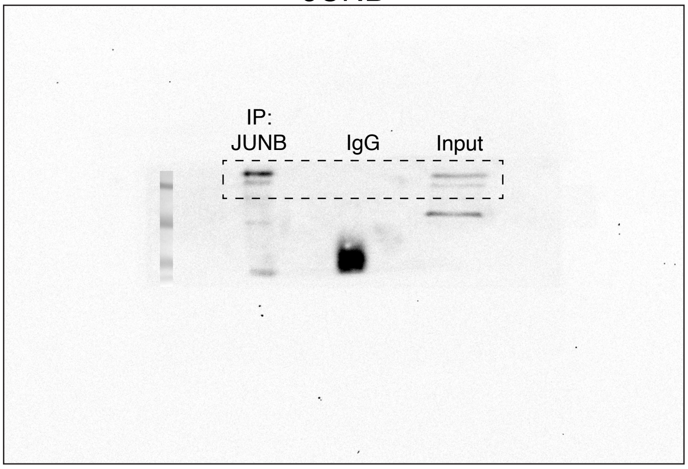

HDAC1

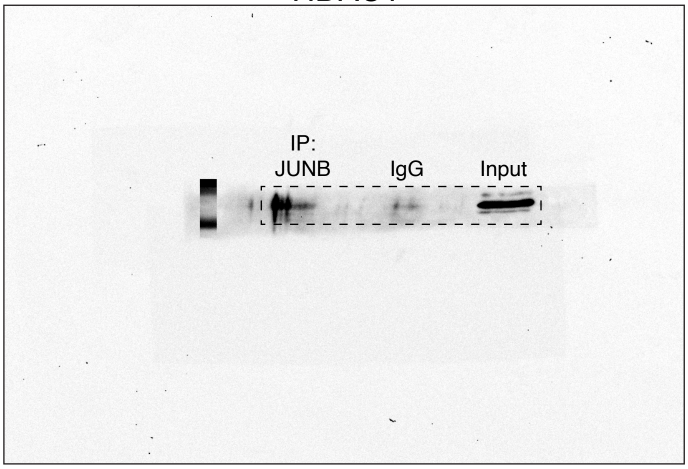

ACTIN

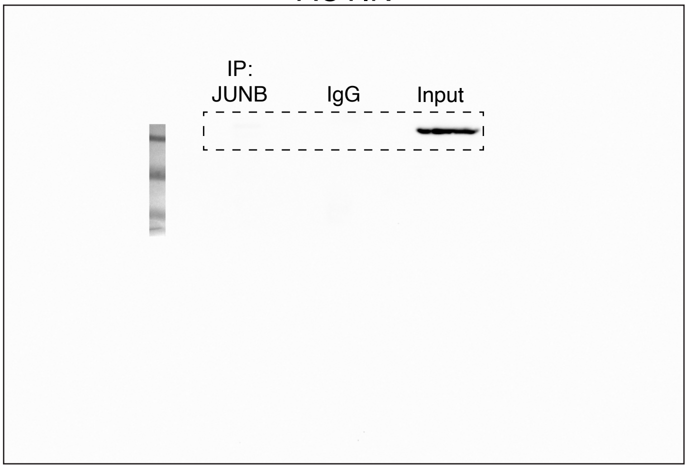

Figure 3m (HPAF-II) **and Figure 3n** (CFPAC1)

JUNB

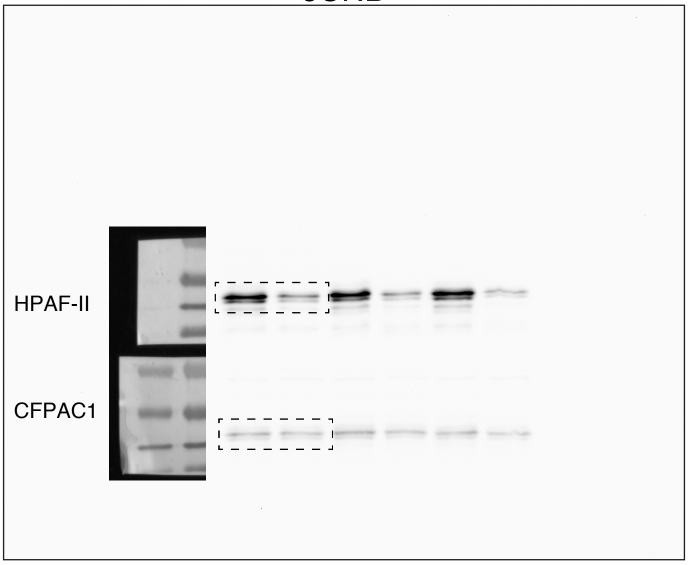

cJUN

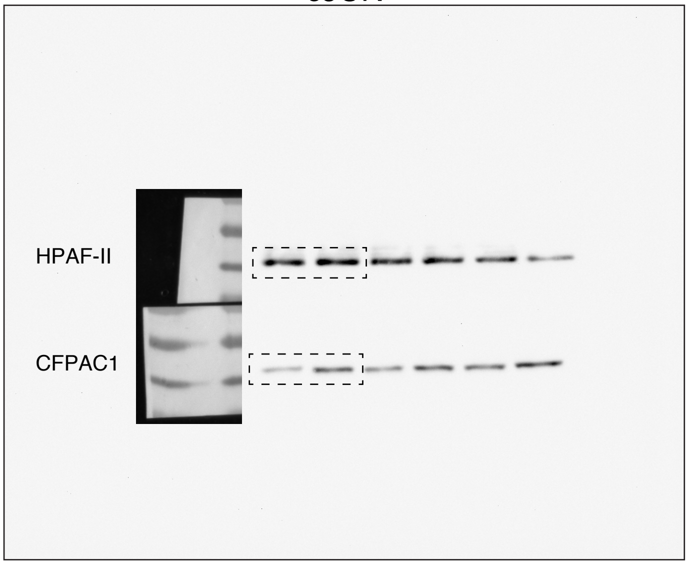

CCL2

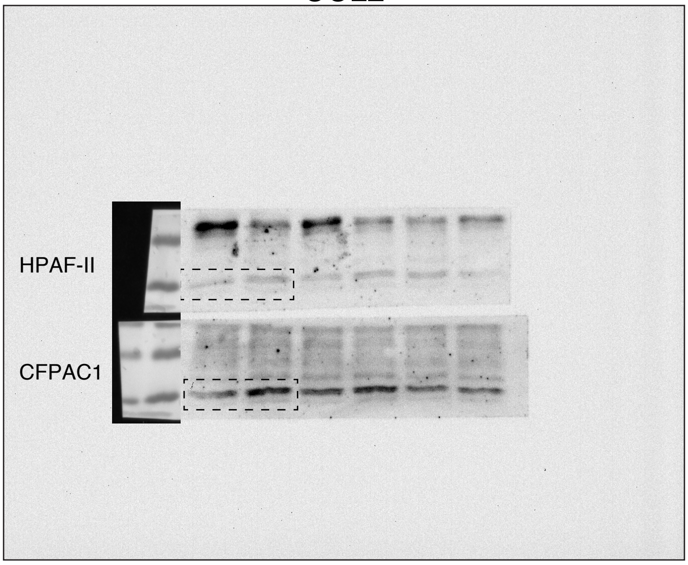

ACTIN

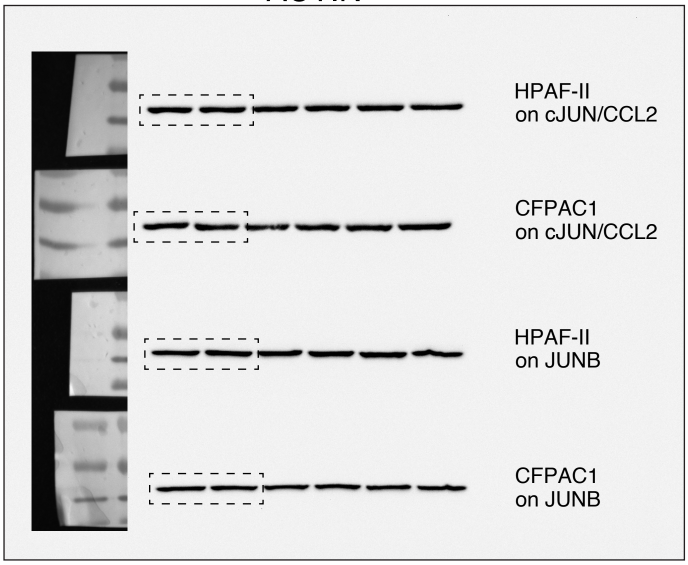

Figure 3p (left, CAPAN1) and **Figure 3q** (right, CAPAN2)

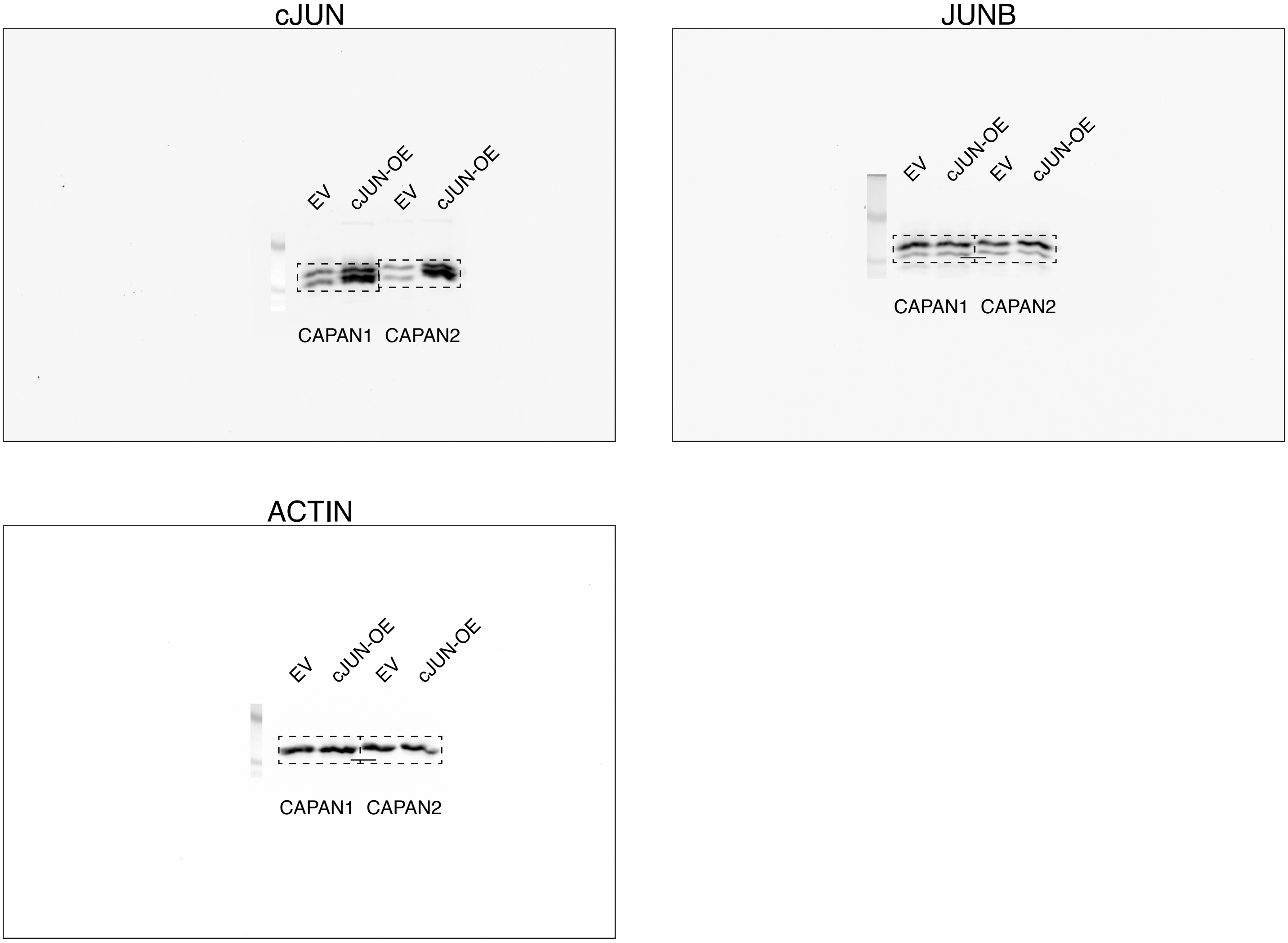

Extended Data Figure 2b

JUNB

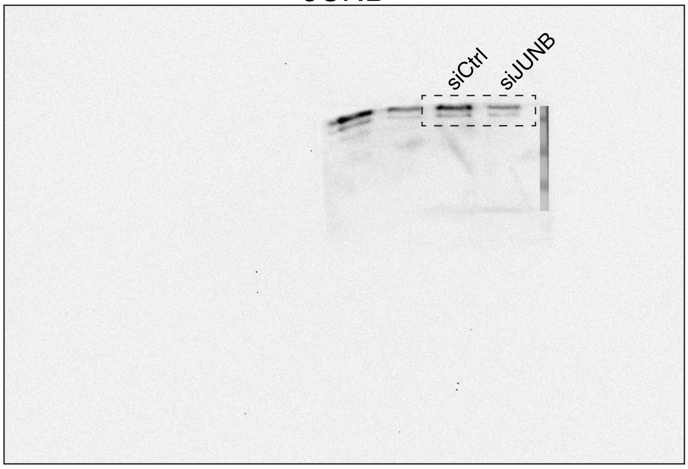

ACTIN

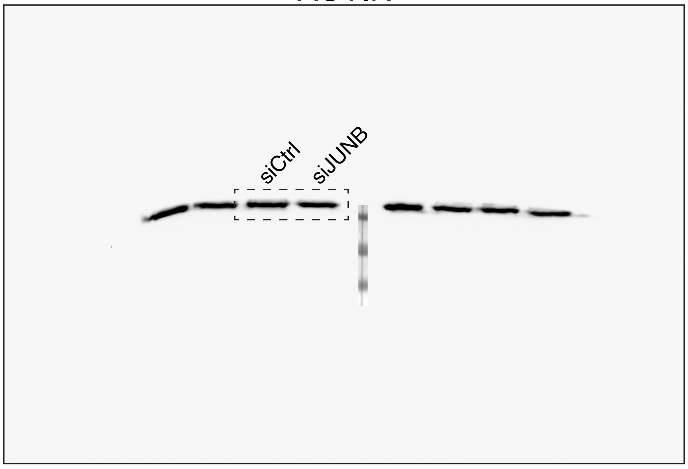

Extended Data Figure 2e

JUNB

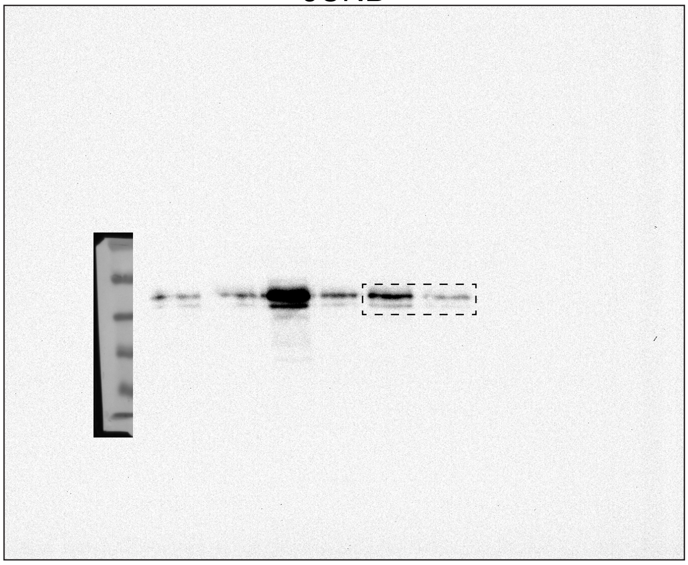

ACTIN

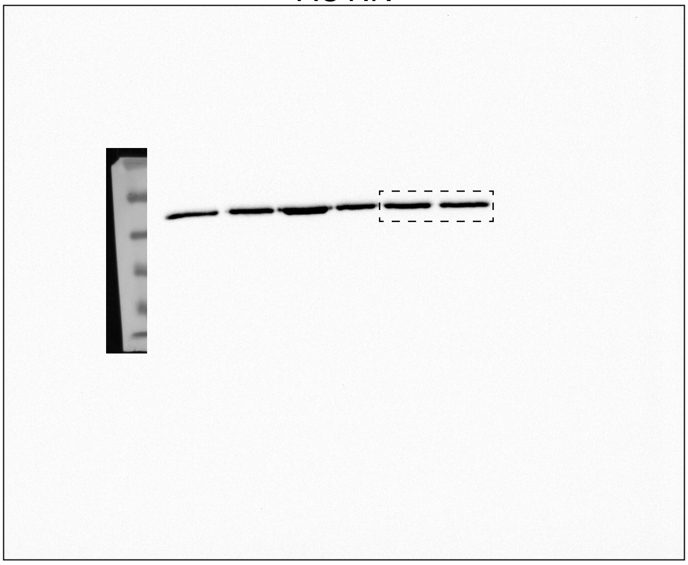

Extended Data Figure 2h

JUNB

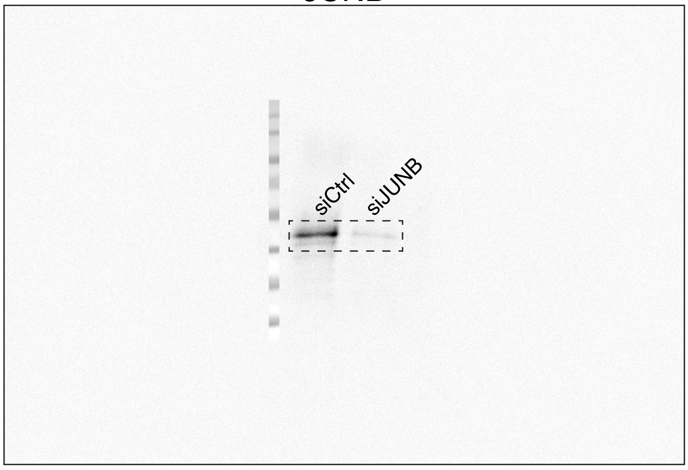

ACTIN

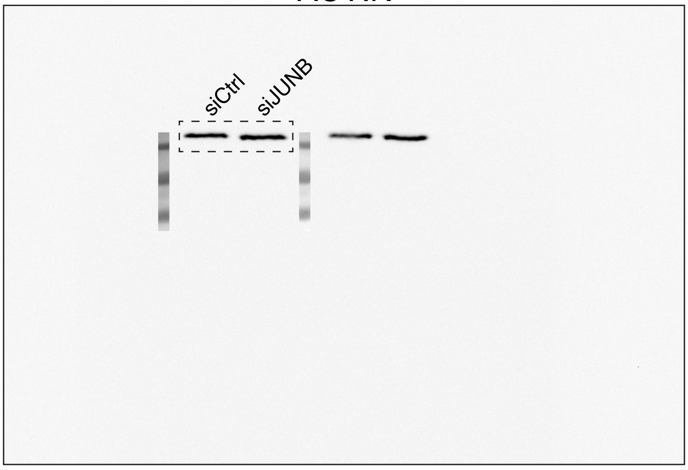

Extended Data Figure 3i

JUNB

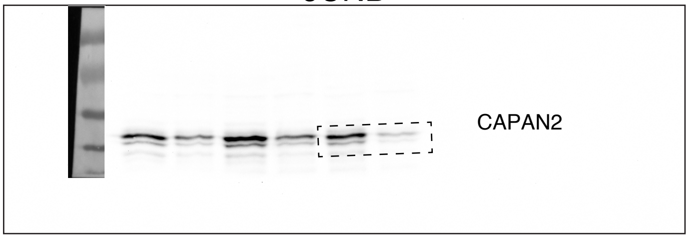

cJUN

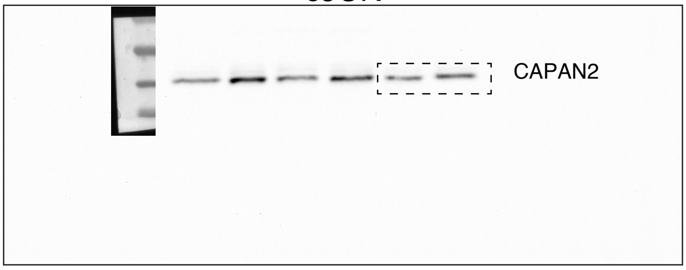

ACTIN

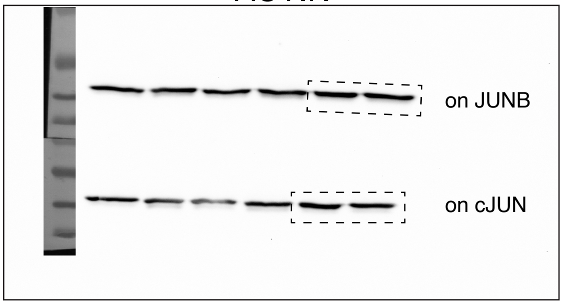

Extended Data Figure 3j

JUNB

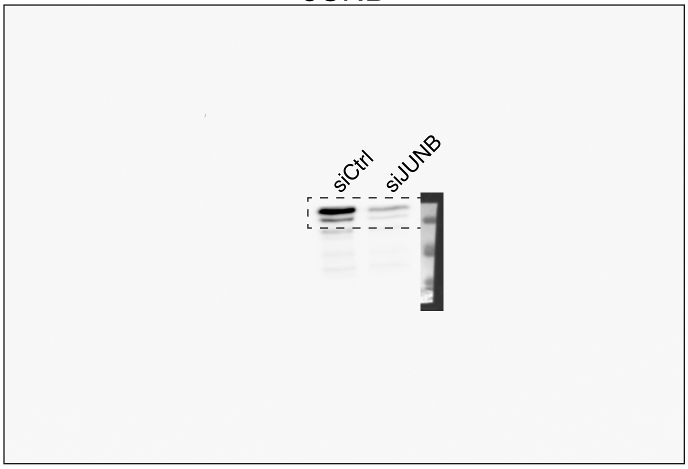

The same marker image was used for JUNB and ACTIN (JUNB).

cJUN

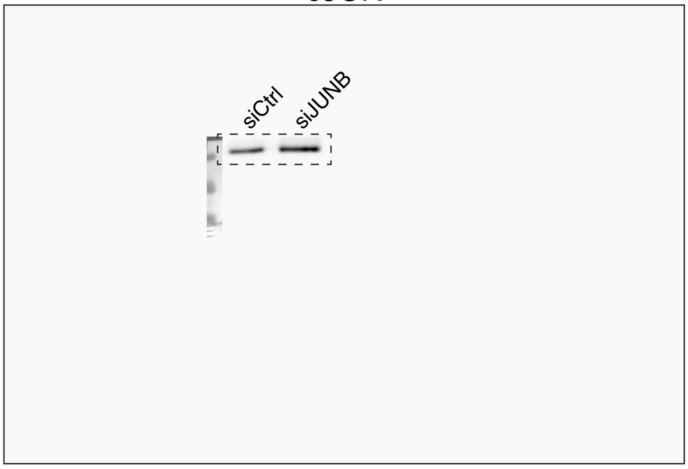

ACTIN (JUNB)

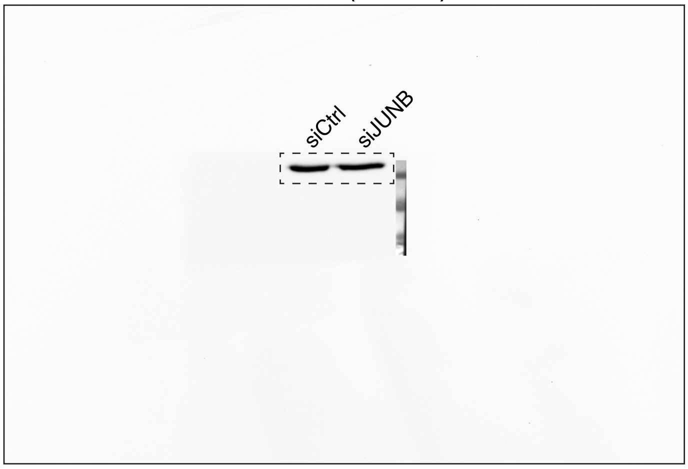

The same marker image was used for JUNB and ACTIN (JUNB).

ACTIN (cJUN)

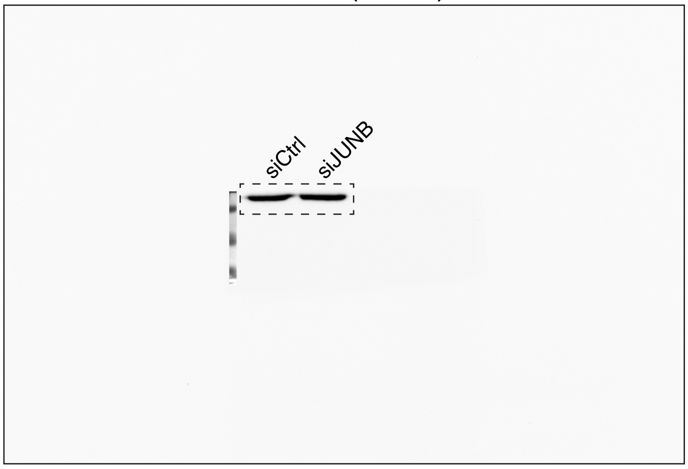

Extended Data Figure 3k

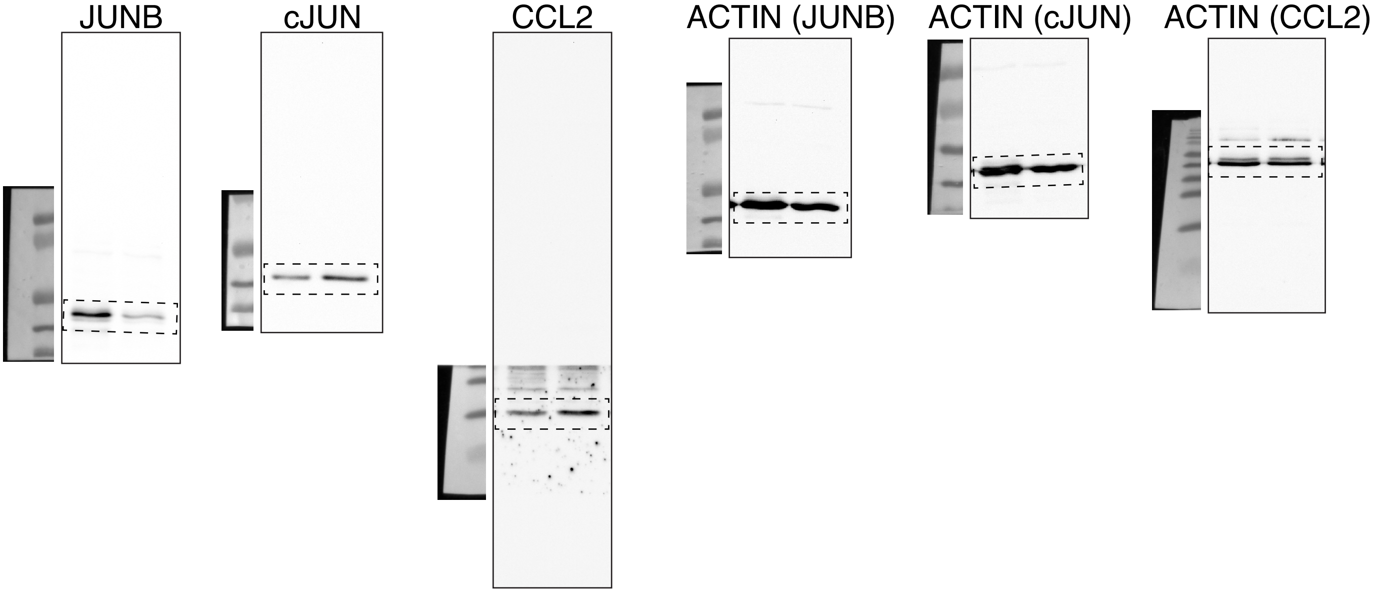

Supplement: Supplementary file 4 — Source Data [file 41467_2024_55330_MOESM4_ESM.zip › Klein-et-al_SourceData.pdf]
